# Using Fish as a Sentinel in Risk Management of Contaminated Sediments

**DOI:** 10.1007/s00244-022-00968-x

**Published:** 2022-12-21

**Authors:** O. Magnus Karlsson, Hannes Waldetoft, Joakim Hållén, J. Mikael Malmaeus, Lars Strömberg

**Affiliations:** 1 IVL Swedish Environmental Research Institute, P.O. Box 210 60, 100 31 Stockholm, Sweden; 2 Swedish Forest Industries Water and Air Management Research Foundation, P.O. Box 555 25, 102 04 Stockholm, Sweden

## Abstract

Sediments polluted by historical emissions from anthropogenic point sources are common in industrialized parts of the world and pose a potential threat to the function of aquatic ecosystems. Gradient studies using fish as a bioindicator are an option to assess the ecological impact of locally polluted areas. This study investigates the remaining effects of historical emissions on sediments outside ten Swedish pulp and paper mills using perch (*Perca fluviatilis*). The aim has been to obtain a general picture of the impact area of local deposits of cellulose fiber-rich sediments containing elevated levels of trace metals, e.g., Hg, and organochlorines, e.g., dioxins. In addition to analyzing contaminant levels in muscle and liver tissue, morphological measures in the fish that constitute biomarkers for health and reproductivity were measured. Another aim was to augment existing historical data sets to observe possible signs of environmental recovery. Overall, the results indicate only a minor elevation in contaminant levels and a minor impact on the fish health status in the polluted areas, which in several cases is an improvement from historical conditions. However, exceptions exist. Differences in the ecosystems' responses to pollution loads are primarily explained by abiotic factors such as water turnover rate, bottom dynamic conditions, and water chemistry. Weaknesses in the sampling methodology and processing of data were identified. After minor modifications, the applied survey strategy has the potential to be a management tool for decision-makers working on the remediation of contaminated areas.

Levels of contaminants in fish and their health status in pollution gradients are a rich source of information for applied environmental science (WHO 1993; van der Oost et al. 2003; Law et al. 2010). Whereas investigations of sediments and other abiotic compartments may provide helpful information on ecosystem *exposure* to hazardous substances (Håkanson and Jansson 1983; Förstner 1989; Jonsson 1992; Tarazona et al. 2014; Apler 2021), investigations of fish have the potential to address the actual environmental *effects* (Södergren 1989; Munkittrick 1992; Sandström et al. 2005). Thus, measurements of contaminant levels in fish and their health status can be used as a complementary tool to assess risks from contaminated sediments on the environment and people's health.

Sediments polluted by metals, organochlorines, and other organic compounds, a legacy from previous unawareness of environmentally hazardous substances, frequently occur outside paper and pulp mills (Pearson and Rosenberg 1976; Håkanson et al. 1988; Jonsson et al. 1993; Kähkönen et al. 1998; Kienle et al. 2013; Hoffman et al. 2017; Apler et al. 2020). The ultimate risk-reducing measure would be removing or capturing the contaminated layers (Peng et al. 2018; Lehoux et al. 2020). However, this is not feasible on a large scale, neither from a technical nor an economic perspective. Direct measures also demand lots of energy, material resources, and areas on land for disposal (Suer et al. 2004). Hence, active measures may be sub-optimal from a sustainability perspective, contradicting other environmental goals, e.g., reducing global warming. The removal of contaminated sediments itself can also be problematic for aquatic life (Baumann and Harshbarger 1998) unless robust protective measures are undertaken. Therefore, society needs tools to distinguish between places where there is a need for risk-reducing efforts to protect aquatic life from areas that, although being contaminated, show small or no signs of being affected and where natural recovery processes occur (Magar and Wenning 2006; Förstner and Apitz 2007; Fuchsman et al. 2014; Fetters et al. 2020).

The environmental effects of emissions from the cellulose industry have, since the 1960s, been an area for environmental research forming the basis for successive mitigation actions to protect aquatic life (Norrström and Karlsson 2015; Ussery et al. 2021). Numerous field studies targeting effects on fish have been conducted in Scandinavia (Sandström et al. 1988; Landner et al. 1994; Förlin et al. 1995; Karels and Oikari 2000), North- and South America (Adams et al. 1992; Munkittrick et al. 1994; McMaster et al. 2006; Chiang et al. 2010; Mower et al. 2011; Barra et al. 2021) and Oceania (Harris et al. 1992; van den Heuvel et al. 2010). For natural reasons, most studies have focused on the effects of present emissions, whereas relatively few studies, with some exceptions (Meriläinen et al. 2001; Hynynen et al. 2004; Arciszewski et al. 2021), have investigated the status of ecological indicators after mill closure. Generally, evident signs of ecosystem recovery have been observed after mill closure or at mills still operating, having improved their environmental performance. However, residual disturbances in terms of eutrophication and toxic effects on fish may persist in some cases (Sandström et al. 2016).

This study conducted field surveys outside ten Swedish paper and pulp mills with well-documented occurrences of polluted fibrous sediments. Some of the mills have been closed for decades, whereas others are still operating, with high demands on environmental performance. The aims were to (1) investigate to what degree the historical emissions from pulping affect today's environmental conditions in the receiving areas concerning pollutant content and health status of the non-migratory, prevalent, and ecologically important fish species *Perca fluviatilis*, (2) incorporate new data into existing data records to conclude the environmental development and possible success of undertaken remedial actions as well as what stressors that remain (3) to identify weaknesses and evaluate the applied sampling strategy, e.g., are the sample sizes and the number of chemical analyses acceptable from a statistical standpoint? In a broader context, the goal is to develop a cost-effective monitoring protocol using fish as a sentinel to guide risk assessments of contaminated sediments in coastal and limnic ecosystems.

## Background

### Chemical Contaminants

From around 1940 until the end of the 1960s, mercury (Hg) preparations were added as a pesticide for mucus control and conservation of wet pulp in most Swedish cellulose industries (Jerkeman and Norrström, 2017). Moreover, at some places along Sweden's coast and inland, chlor-alkali plants have been located next to the cellulose industries to produce lye and chlorine gas for boiling and bleaching chemical pulp. In that process, significant Hg emissions to air and water were also generated through residual emissions of graphite sludge. The total discharges of Hg from the Swedish cellulose- and chlor-alkali industries have been around 500 tons (Jerkeman and Norrström, 2017). The usage of Hg was banned in Sweden in 1968. As a result, emissions from the pulp- and paper industry were sharply reduced around 1970.

Traditionally, the potentially hazardous metals such as arsenic, lead, cadmium, copper, chromium, nickel, and zinc are measured in Swedish environmental monitoring. The primary source of these metals within the cellulose industry was, and still is, the raw wood material since the trees take up metals from the soil and are exposed to atmospheric deposition. Metals generally occur in low levels in wastewater from the process, usually not being reduced in the effluent treatment. Therefore, handling large amounts of wood produces significant emissions (Sandström et al. 2016). Moreover, in the past, the production of sulfuric acid by roasting sulfur silica for sulfite pulp production generated pyrite-ash contaminated by metals that may have been released into the biosphere (Baragaño et al. 2020; Apler 2021).

Pollution by persistent organics has also occurred in areas where pulp mill effluents have been discharged. Chlorinated dioxins and furans (PCDD/Fs) were previously inadvertently formed in manufacturing bleached chemical pulp when elemental chlorine was used in the bleaching process (Swanson et al. 1988). Increased closure and the transition to bleaching with chlorine dioxide in the late 1980s and early 1990s significantly reduced emissions of chlorinated substances, and the dioxin formation ceased (Berry et al. 1991; Strömberg et al. 1996). In addition, which also applies to other globally dispersed pollutants, precipitation of long-range air-borne emissions over the forests where the raw material grows can accumulate dioxins in the bark of the trees (Salamova and Hites 2010). Therefore, a possible source of dioxins is run-off from wood storage and debarking. Atmospheric deposition over the catchment area of the mill's raw water supply (Josefsson et al. 2011) and over the effluent-receiving water bodies is another potential source.

Neither of the chlorinated compounds PCBs, DDT, and HCB have been used as a specific auxiliary or additive chemical in the pulping process. However, PCBs have generally had extensive use in society, such as additives in hydraulic and transformer oils and sealants in building structures (Breivik et al. 2002). DDT has been used for insect control at the timber stores in the forest and may have accompanied the raw material into the industrial areas. The use of DDT in Sweden was banned in 1969. HCB was inadvertently formed in the chlor-alkali process. Atmospheric deposition of these compounds over the forests where the raw wood material has grown may also, like PCDD/Fs, have contributed to a presence in the bark (Salamova and Hites 2010).

### Fish in Environmental Monitoring

The non-migratory, spring-spawning fish species European perch (*Perca fluviatilis*) is frequent in Scandinavia's brackish and freshwater ecosystems. Being relatively sedentary (Hansson et al. 2019), perch has for decades been used for environmental monitoring purposes (Sandström et al. 2005). The fall is the period for active gametogenesis of perch and the standard sampling period in the Swedish national monitoring of contaminant levels and health status (SMNH 2012). For contaminant monitoring, perch with a length of 15–20 cm is usually used, whereas the effect monitoring programs often use fish within the length interval of 20–25 cm (SEPA 1997) but also smaller individuals divided into length classes (Sandström and Neuman 2003).

Reflections on long-term records of surveys in ecosystems may provide valuable information for environmental management (Arciszewski et al. 2021; Ussery et al. 2021). An evaluation of about fifty fish health surveys in the Swedish cellulose industry receiving waters conducted between 1985 and 2015 (Sandström et al. 2016) found that there has been a good recovery of fish health in most receiving waters compared to conditions during the 1980s and 1990s. In general, biochemical health measures had responded well to process improvements in the mills. In contrast, morphometric measures seem to have had a longer response time, and residual effects on mainly reproductive measures were demonstrated in some receiving waters in recent years. This, combined with experience from the Canadian EEM program (Lowell et al. 2005) and the practical benefits of focusing on morphological measures, guided the final choice of biomarkers.

## Materials and Methods

### Study Areas

**Fig. 1:**
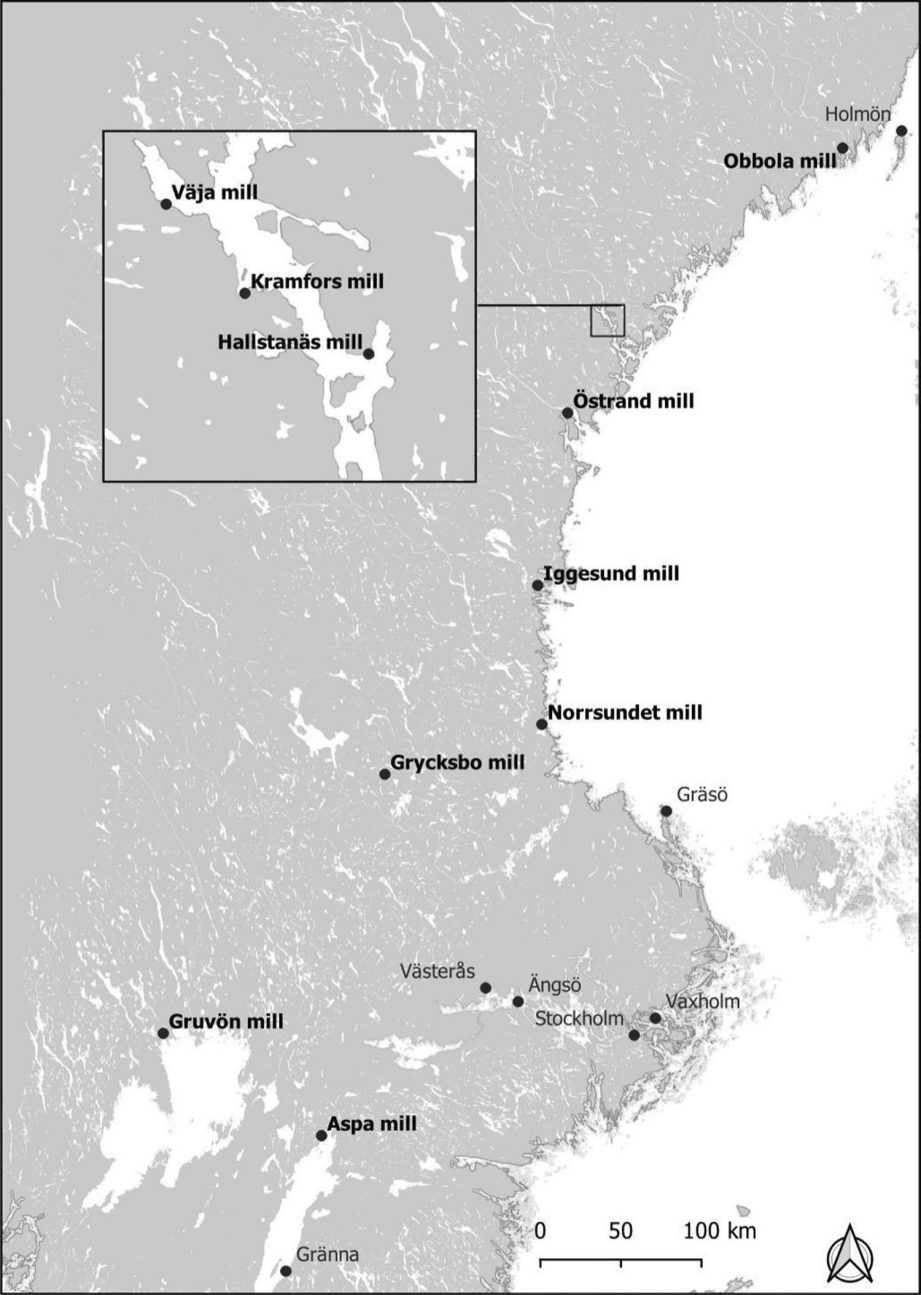
Mill study areas and comparison areas

**Fig. 2:**
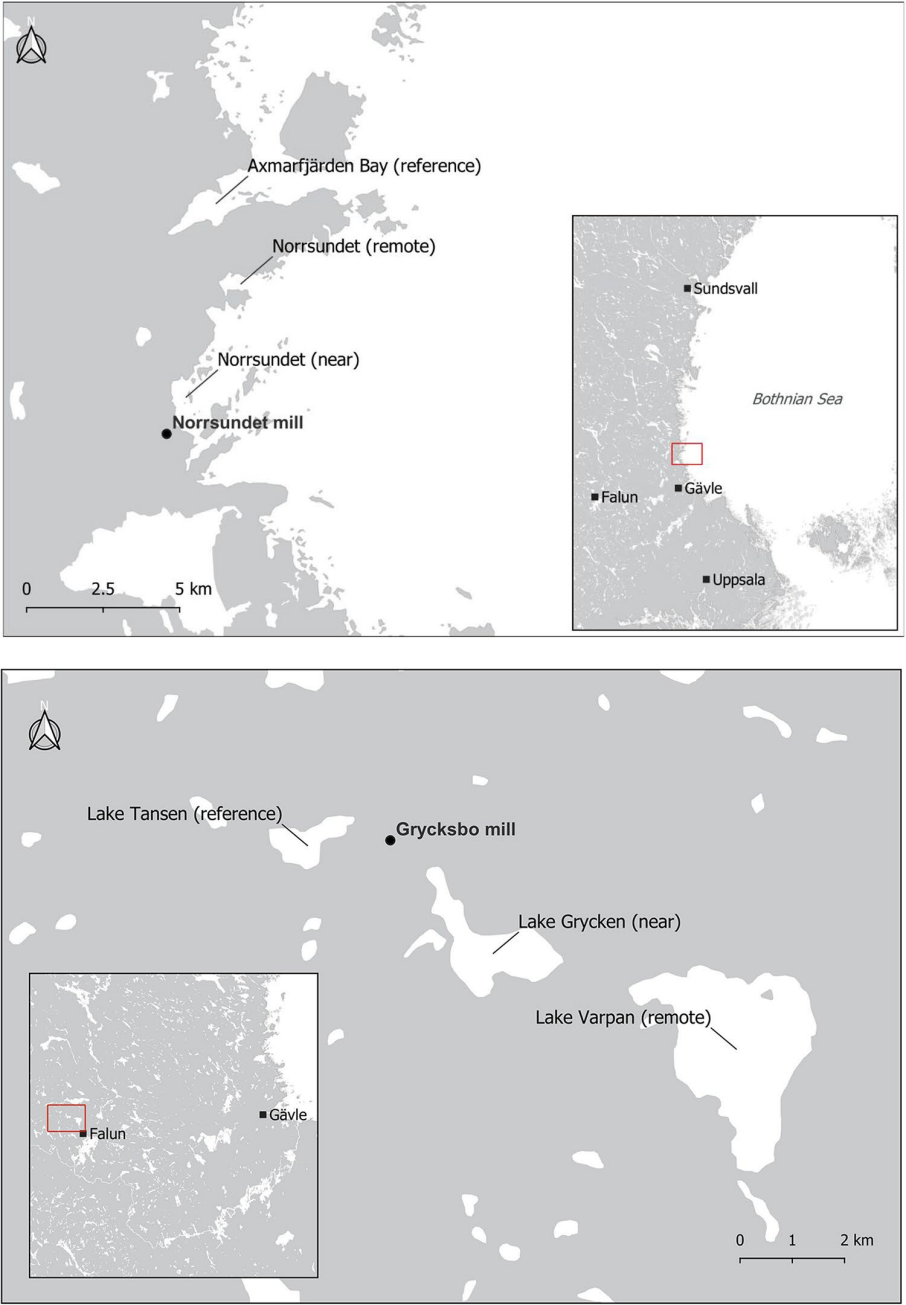
Examples illustrating the concept of near and remote receiving areas and reference areas at Norrsundet (upper) and Grycksbo (lower)

The survey included areas around ten Swedish mills adjacent to coastal areas, large lakes, and a small forest lake (Fig. 1; Table 1). The production has ceased at Hallstanäs in 1976, Kramfors in 1977, and Norrsundet in 2008, while the others are still active. However, the production processes may have changed considerably since the emissions of pollutants took place, in most cases going back to the 1970s and earlier. Some mills, especially Norrsundet, have a data record of regularly recurring surveys since the 1980s, while others have only been surveyed sporadically. The principle for selecting sites within each area, following previous Swedish sampling strategies (SEPA 1997), is shown in Fig. 2. A sampling point named "near" (near receiving site) was placed near the mill's discharge point and identified sediment contaminants. Within 5–10 km from the mill but still within reach of the wastewater plume, another sampling point, called "remote" (remote receiving site), was placed. Upstream, or at a sufficiently large distance from the mill to be considered unaffected by the industrial emission, a sampling point designated "ref." (reference site) was placed. In Waldetoft et al. (2020), detailed maps from each study site are available. Results from seven other Swedish lake- and coastal areas sampled simultaneously (Fig. 1) were also included for comparison.

**Table 1:** Production and characteristics of the receiving water areas at the study sites

Mill	Production type	Status	Receiving water area	Water turnover time	Water phosphorus concentration (Tot-P, µg/l)
Obbola	Unbleached kraft pulp, recycled fiber pulp, paper	Active	Coastal area	< 1 week	11
Väja	Unbleached kraft pulp, paper	Active	Coastal area	1–2 weeks	10
Hallstanäs	Stone groundwood pulp	Closed 1976	Coastal area	1 month	8
Kramfors	Unbleached sulfite pulp	Closed 1977	Coastal area	1 month	8
Östrand	Bleached kraft pulp, CTMP pulp	Active	Coastal area	1–2 weeks	9
Iggesund	Bleached sulfite and kraft pulp, paperboard	Active	Coastal area	1–2 weeks	17
Norrsundet	Bleached kraft pulp	Closed 2008	Coastal area	1–2 weeks	12
Grycksbo	Bleached sulfite pulp and paper	Active*	Small lake	2 months	12
Gruvön	Bleached kraft pulp, paper, and paperboard	Active	Large lake	1–2 weeks	8
Aspa	Bleached and unbleached kraft pulp	Active	Large lake	< 1 week	3

^*^pulp production ended in 1977

### Field Sampling and Dissection

The fieldwork was carried out from 2017 to 2019. Sampling of fish, length 15–20 cm, took place from late August to early October. Fish were captured using gill nets (18–25 mm mesh size) set overnight. After the sampling, the fish was frozen on-site and transported to IVL Swedish Environmental Research Institute's laboratory in Stockholm. In total, 878 individuals were sampled and examined from the ten study areas (Table 2).

**Table 2:**
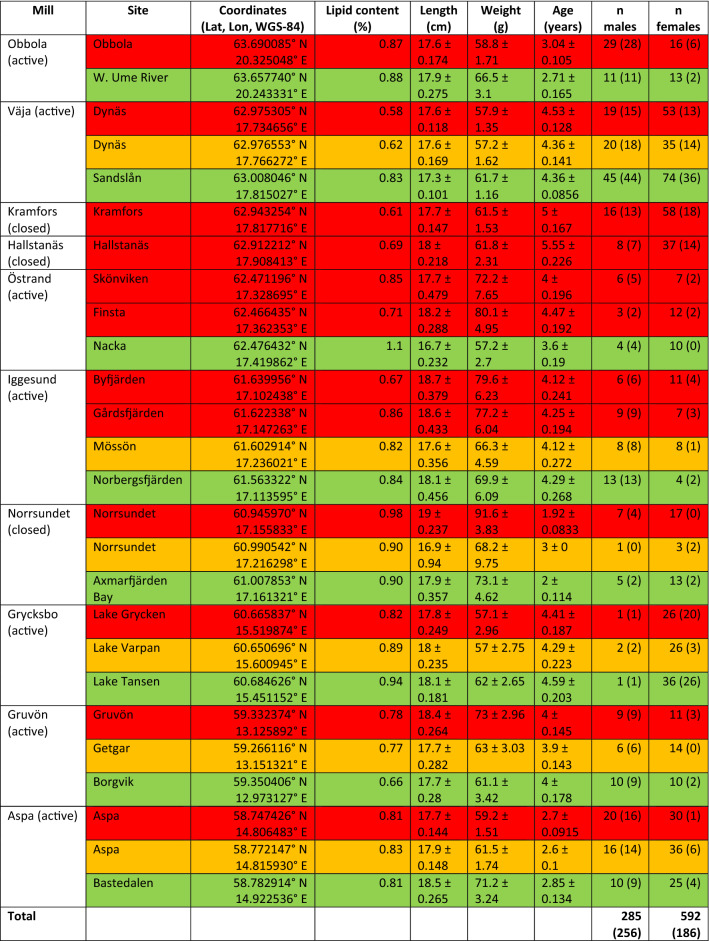
Descriptive statistics (mean ± standard error) of sampled fish. Red = near receiving site, yellow = remote receiving site, green = reference site. The number of sexually mature individuals in parenthesis

For each individual, the condition factor (CF: 100 × weight (g)/length^3^ (cm)), gonadosomatic index (GSI: 100 × gonad weight (g)/somatic weight (g)) and liversomatic index (LSI: 100 × liver weight (g)/somatic weight (g)) were calculated. In addition, an assessment of growth (cm/year) was made based on back-calculated length (Sandström et al. 1988). Also, the proportion of sexually mature individuals (SM) was calculated. Individuals with GSI ≥ 1 were considered sexually mature. After dissection, three pooled muscle and liver tissue samples were prepared from each fishing site. Each pooled sample contained an equal amount of tissue (about 100 g) from four to ten, most often eight, fish individuals.

### Chemical Analyses

Standard analytical methods were applied; for Hg, US EPA 1631, other trace metals, SS-EN 13,805:2014, PCDD/F, US EPA 1613, PCB, DDT, and HCB, SS-EN 16,167:2018, EN ISO 6468:1996. Organochlorines and Hg were analyzed in muscle tissue, whereas the other trace metals were analyzed in liver tissue. Detection levels are shown in Table 3.

**Table 3:** Applied methodology

Sampling time	September, during active gametogenesis
Fish species	European perch (*Perca fluviatilis)*
Fishing method	Overnight gill nets 18–25 cm mesh size
Fishing effort	≥ 30 individuals between 15 and 20 cm in length from each study site
Morphological measures and indices	Length, total weight, condition factor (CF), liver-somatic index (LSI), gonad-somatic index (GSI), growth, and degree of sexual maturity
Sample preparation	Three pooled samples of 10 individuals from each site
Investigated pollutants	Analyses in muscle tissue: Hg (mg/kg w.w., 0,03*), PCDD/Fs + dl-PCB (pg TEQ/g w.w., 0.10*) PCB_7_ (ng/g w.w., 0.10*), HCB (ng/g w.w.,0.07*) DDT (ng/g w.w. 0.07*), Fat content (0.1%*). Analyses in liver tissue: As (mg/kg d.w., 0.003*), Cd (mg/kg d.w., 0.001*), Cr (mg/kg d.w.,0.01*) Cu (mg/kg d.w., 0.01*), Ni (mg/kg d.w., 0.04*), Pb (mg/kg d.w.,0.003, *), and Zn (mg/kg d.w.,0.07*), *Limit of detection (LOD)

### Evaluation

A linear mixed effect model was used to assess differences in mercury and cadmium levels between near receiving, remote receiving, and reference sites for all mills at once and between different environmental types (i.e., coastal, estuary, and lake (Table 1)). The contaminant was the response variable. The type of site, environmental type, and the interaction between these were the explanatory variables. The random effects were to which mill and to which site an observation belongs. Tukey's pairwise comparison was used as a post hoc test.

One-way ANOVA was used for evaluating PCB levels within sites. A sample size of three samples per site is small, but on the other hand, variances are reduced when pooling samples. A simulation showed that ANOVA on pooled data, compared to individual data, leads to inflated *p*-values, but differences of ecological relevance can be detected. Thus, it was decided to conduct and report the results of the tests.

Using linear regression, statistical evaluation of the CF, LSI, GSI, and growth at age was performed for each mill. Each morphometric index was the response variable. The explanatory variables were *site* (near receiving/remote receiving/reference) and *gender* (male/female). Gender was included as a control variable to account for differences between the sexes. For the analysis of GSI, only sexually mature individuals were included. The regression for growth at age was performed on log-transformed values with the logarithmized age of the fish as a control variable. Dunnett's test was used as a post hoc test.

The test for differences in sexual maturity (SM) was performed using a logistic regression model with gender status (sexually mature/not sexually mature) as the response variable and *site* (near receiving/remote receiving/reference) and *length* of the fish (cm) as explanatory variables. All analyses were performed in R (R Core Team 2020), and an example code is available in Waldetoft et al. (2020).

In addition to evaluating statistical significance, the concept of critical effect size (CES) was used (Munkittrick et al. 2009). The idea was developed in the Canadian EEM program for CF, LSI, and GSI (Lowell et al. 2003). CES acts as an identifier of a minimum difference between receiving water and reference sites regarded as potentially unacceptable. CES for CF, LSI, and GSI is  ± 10%, ± 25%, and ± 25%, respectively. For example, if LSI in a receiving water site is more than 25% larger than in the reference site, CES is said to be exceeded. In the Canadian EEM program, if CES is exceeded at a mill for two consecutive surveys, it acts as an identifier that either more focused monitoring to assess the magnitude of the effect or an investigation of the cause of the effect is needed.

A meta-analysis was performed to assess whether overall response patterns were present for any morphological index. The model used was a meta-analytic mixed effects model (e.g., Raudenbush 2009). A two-stage approach was used (Burke et al. 2017), in which coefficients and variance–covariance matrices from regressions on each mill were used in the meta-analytic mixed effects model. The meta-analytic model was fitted using the metafor-package in R (Viechtbauer 2010). The fixed effect was the model coefficients, and each coefficient was allowed to vary randomly for each mill by specifying the random effects as random intercept and slope.

The time trends of Hg and PCDD/F levels in fish were assessed statistically using linear regression with the natural logarithm of Hg content as the response variable and sampling year as the explanatory variable.

### Areal and Temporal Comparisons, Normalization

Parallel with the fish surveys in the cellulose industry receiving waters 2017–2019, IVL Swedish Environmental Research Institute has conducted fish surveys using the same methodology in other inland and coastal waters in Sweden to address contaminant levels. Data from these surveys have been used for comparisons (denoted in figures as "comp. areas"). In addition, data from previously published articles, reports from regional environmental monitoring, and environmental assessments performed according to the permitting processes for individual mills (Södergren 1989; Lundgren et al. 1991; Heinemo 2001; Olsson et al. 2005; Karlsson and Malmaeus; 2012; Sandström et al. 2016; SEPA 2022) were used to evaluate time trends in pollutant levels.

Due to bioaccumulation, Hg levels in fish are correlated to fish age and size (Olsson 1976). Therefore, to improve the comparability of Hg content among fish of different sizes and sites in space and time, observed Hg levels were standardized to correspond to a 300-g perch based on an empirically supported transfer function (Åkerblom et al. 2014).

Most organochlorines are lipophilic and therefore correlated with the fish's lipid content. To enable comparisons between different species and different parts of the fish, levels of lipophilic substances in the European Union are normalized to 5% lipid content (European Commission, 2014). Perch is a lean fish where lipid content typically varies between 0.5 and 1%. In this study, a typical value for the perch lipid content was set to 0.8%, and measured levels were normalized to 5% lipid content, i.e., multiplied by a factor of 6.25. Toxic equivalence (TEQ) for PCDD/F and dioxin-like PCBs (dl-PCB) was calculated using congener-specific toxic equivalence factors (TEFs) reported by the World Health Organization (van den Berg et al. 2006). When the PCDD/F levels were below detection limits for all congeners, the average medium bound for all samples with no detectable PCDD/Fs was used to calculate TEQ. An overall summary of the applied methodology is given in Table 3.

## Results and Discussion

### Levels of Contaminants

#### Metals

**Fig. 3:**
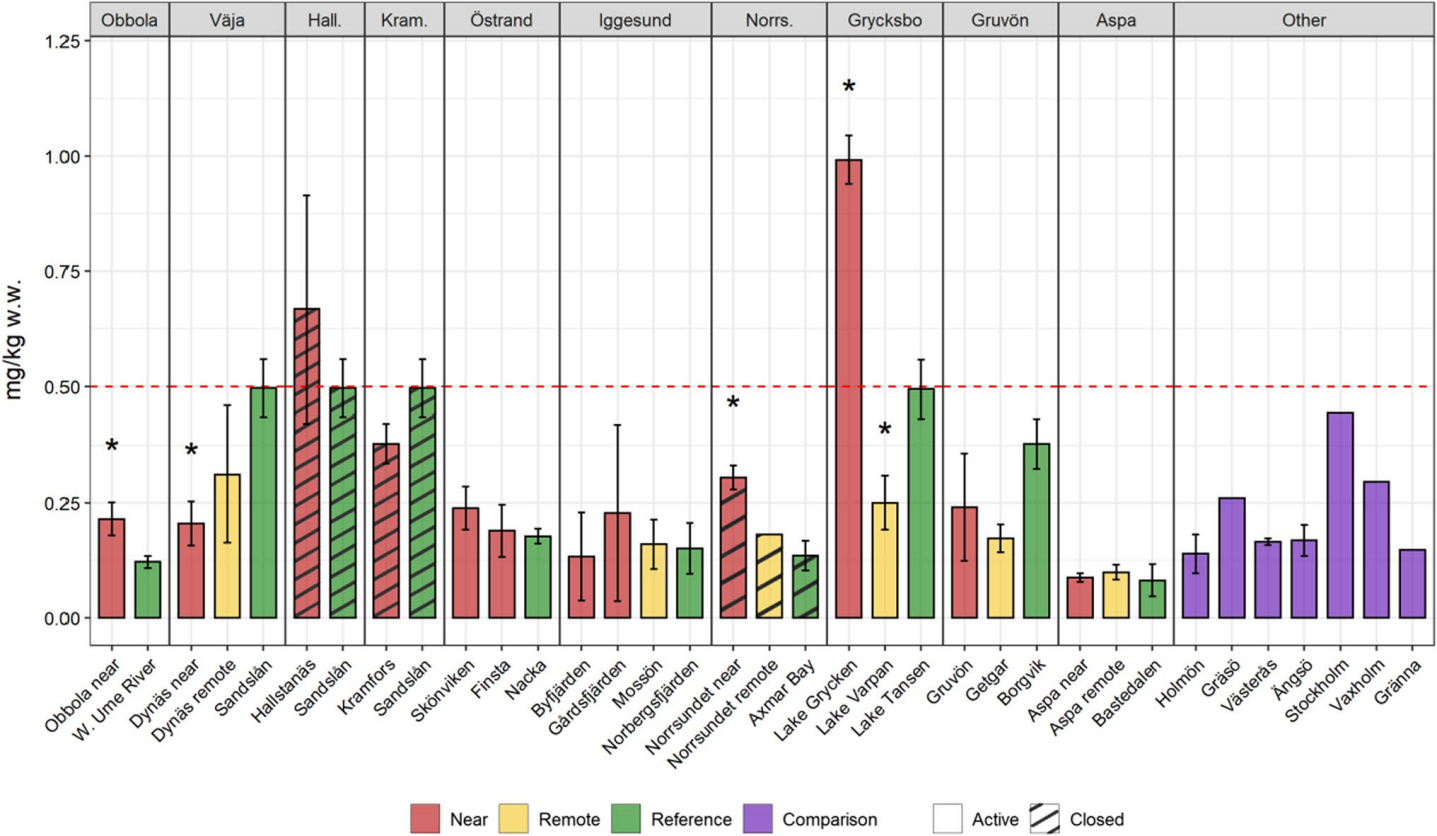
Levels of Hg in pooled samples of 5–10 perch individuals (15–20 cm length) caught between 2017 and 2019. Error bars show 1.96*SE (SE = standard error). Stars indicate significant differences toward reference (5% significance level). Red line at 0.5 mg/kg ww = marketing limit value within the European Union (EC 1881/2006)

**Fig. 4:**
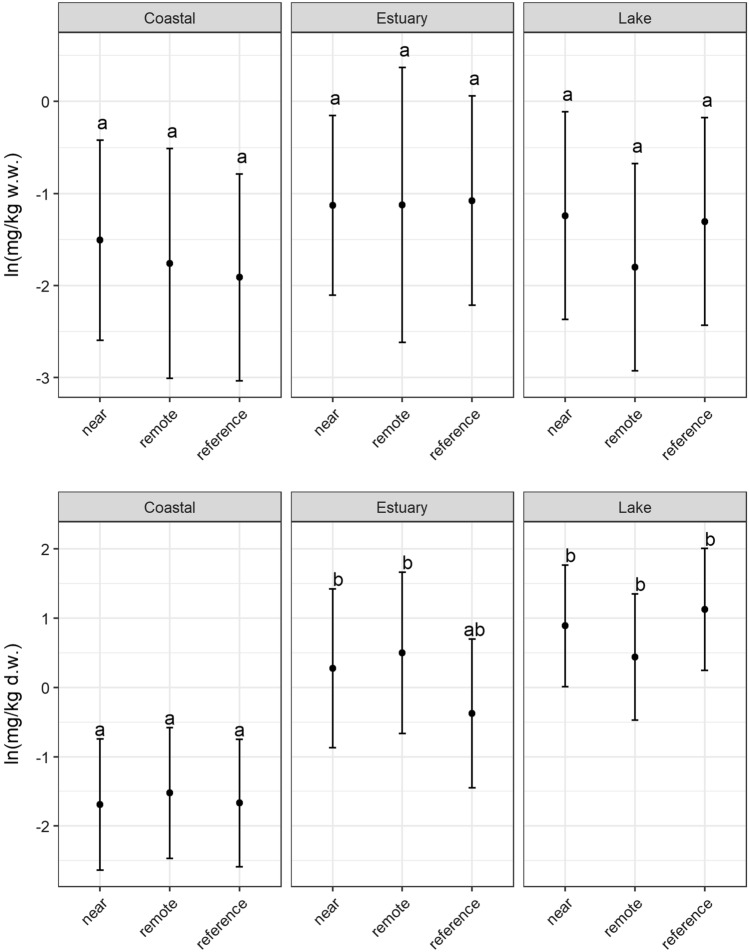
Average Hg (upper)) and Cd (lower) levels in perch from near receiving, remote receiving, and reference sites categorized in the environmental types Coastal, Estuary, and Lake. The same letter indicates a non-significant difference (5% significance level). Values were log-transformed before analysis

Concerning Hg levels, differences between sites are found at specific mills (Fig. 3). In two areas, Hallstanäs and Lake Grycken, Hg levels exceeded the limit value for fish marketing within the EU. There are well-documented fiber banks with high Hg content in both areas. It is also noteworthy that in the Grycksbo system, the levels in the upstream reference lake are approximately at the marketing limit. However, there is no statistical evidence for overall higher Hg in perch from near and remote receiving sites in relation to reference sites for each environmental type (Fig. 4a).

**Fig. 5:**
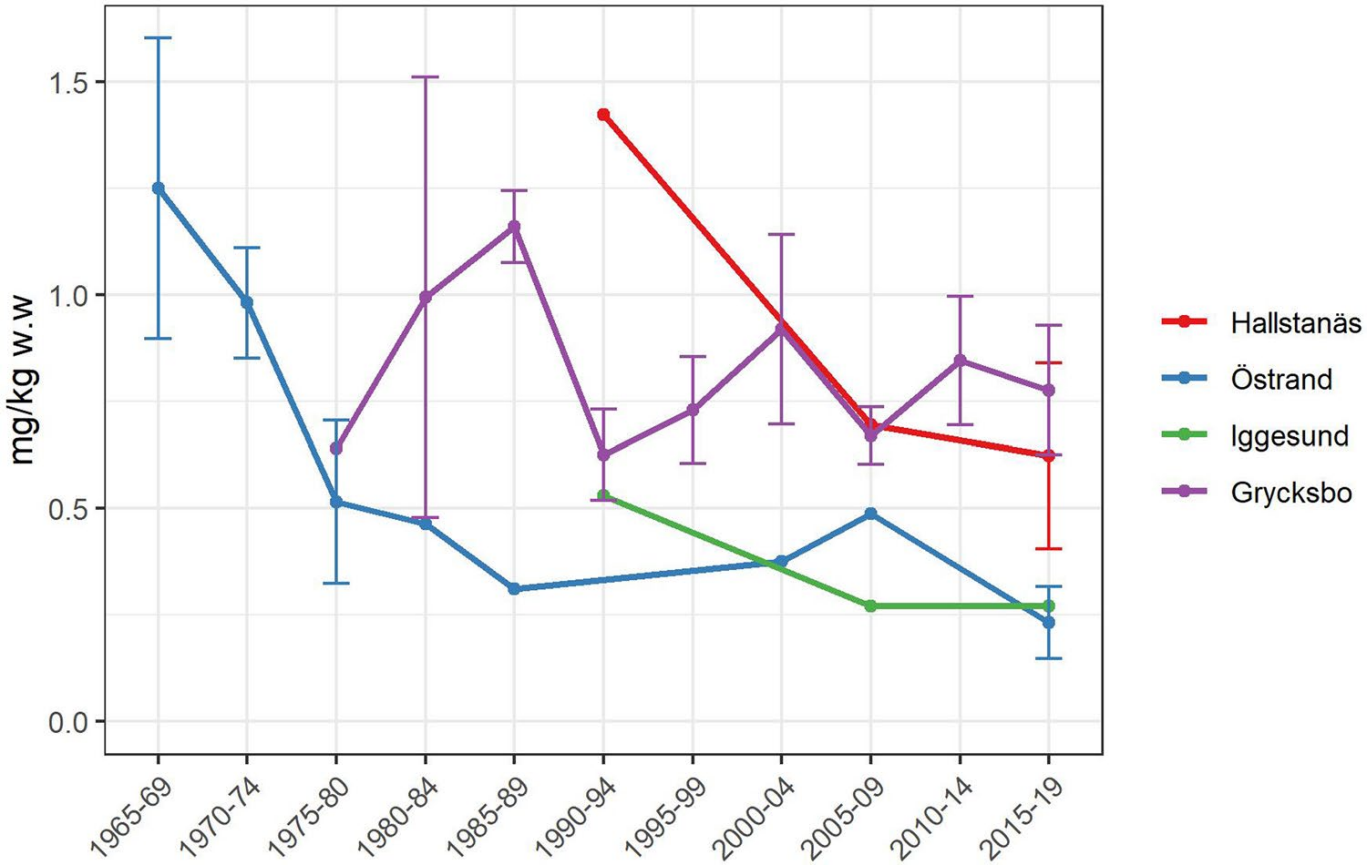
Time trends of Hg levels in perch in the receiving waters of four mills per 5-year period from 1965 to 2019. Historical data from regional environmental monitoring programs. Error bars indicate standard deviation (SD)

The general trend in fish from Swedish waters is that Hg levels have decreased over the 50 years of environmental monitoring (Johnels et al. 1967; Lindeström, 2001; Åkerblom et al. 2014). Figure 5 shows time trends of Hg levels in fish from study sites where such data were available. At Östrand, a significant decreasing time trend was found (*p* < 0.05). At Grycksbo, the time trend was non-significant (*p* > 0.05). Hallstanäs and Iggesund were not examined statistically due to small sample sizes.

Decreasing Hg levels in fish over time have also been reported in studies of the Great Lakes of North America (Blukacz-Richards et al. 2017) and other inland and coastal waters of the USA, Canada, and Australia (Munthe et al. 2007). However, recent studies have also indicated increasing levels (Miller et al. 2013; Gandhi et al., 2014). The reason is unclear, but confounding factors may be increased long-range atmospheric Hg depositions and changes in land use around the sampled water bodies. In summary, a trend toward declining Hg levels is clear but not unambiguous. Environmental factors affecting methylation, bio-dilution, uptake, sediment burial, and how the Hg was released (inorganic form or as directly bioavailable phenylmercury) can explain some of the differences. Plausible explanatory factors for the observations (Fig. 3**)** are, e.g., the lakes in the Grycksbo system, are low-productivity humic lakes, which is a general risk factor for elevated Hg levels (Lindqvist et al. 1991; Sonesten 2003). At Hallstanäs, Hg was discharged in a directly bioavailable form (Heinemo 2001). In the Aspa receiving area (Lake Vättern), high levels of zinc counteract the uptake of Hg (Lindeström et al. 2002).

Cadmium (Cd) is an example of a substance whose bioavailability, to a large extent, is controlled by water chemistry, e.g., salinity and the presence of chloride ions (WHO 1992). The Cd levels in the investigated freshwater lakes around Grycksbo, Gruvön, and Aspa and the low-saline estuary areas in the vicinity of Obbola, Väja, Hallstanäs, and Kramfors were significantly higher than those in the more brackish coastal waters outside Östrand, Iggesund, and Norrsundet (Fig. 4b). In contrast to the differences between the environmental types, there were no significant differences between the different site types (near, remote, and reference). Cd is thus an example of a substance where comparisons between areas must be made with caution, where it is of utmost importance to have reference areas of the same environmental type for comparison, and where levels in fish in absolute numbers cannot be used for risk assessment of contaminated sediments.

**Fig. 6:**
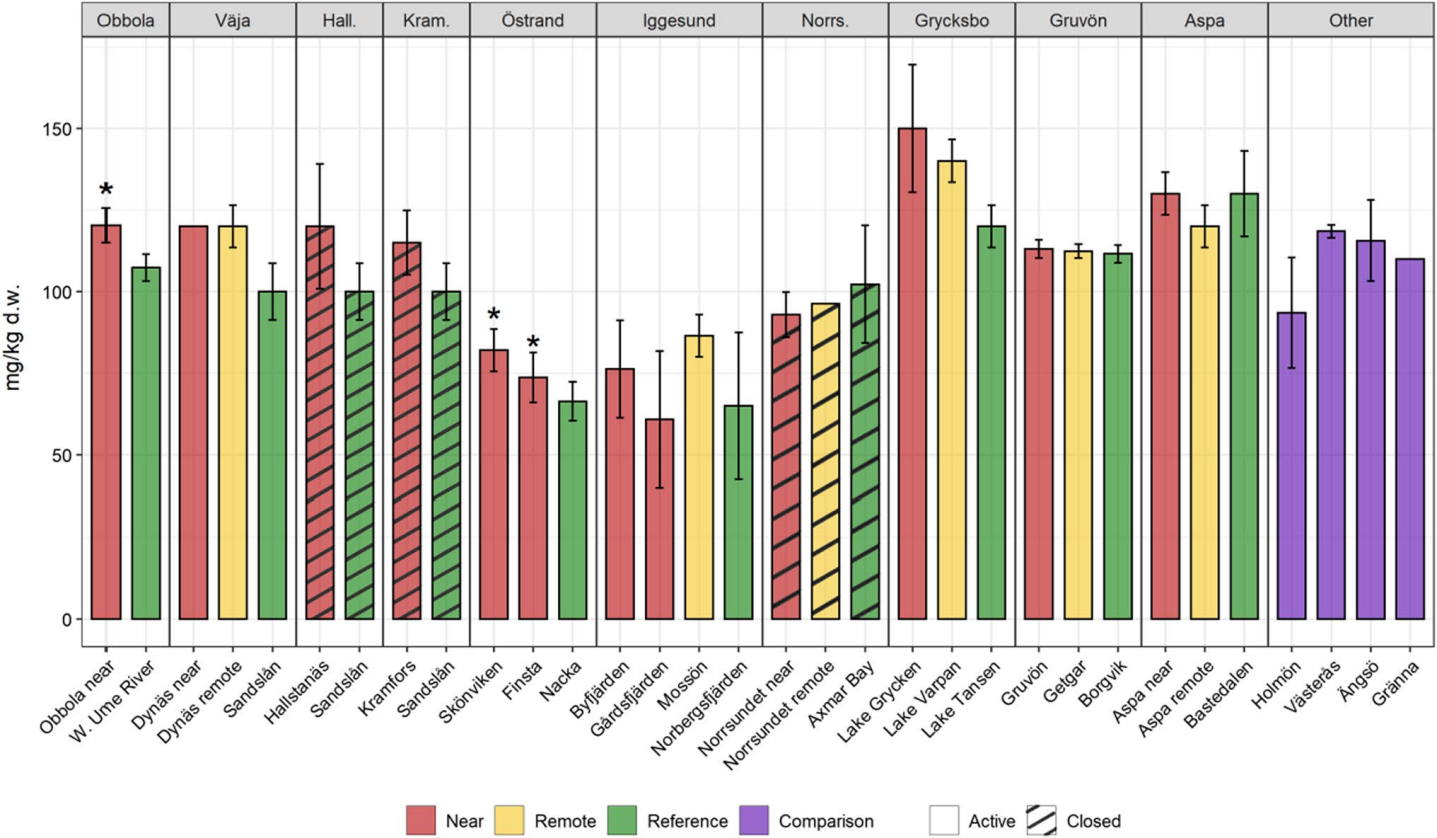
Zn levels in perch from the study sites. Error bars show standard errors (SE). Stars indicate a significant difference toward reference (5% significance level)

In contrast, other metals, e.g., zinc (Zn), show less variation both within and between the study areas **(**Fig. 6). Zn is an essential substance for all organisms, which means that fish have a more prominent ability to regulate the metal themselves. Thus, Zn is an example of a substance where measured levels in fish cannot be used for risk assessments of contaminated sediments.

Raw data for measured levels of the other trace metals (As, Cr, Cu, Ni, Pb) are presented in Appendix A and evaluated in Waldetoft et al. (2020). Experimental studies have indicated that metals bound to fiber sediments generally have low bioavailability (Apler et al. 2018; Frogner-Kockum et al. 2020). Environmental factors, e.g., water chemical properties and mineralizations in the catchment area bedrock, can play a more significant role than the exposures to locally polluted sediments for the metal content in fish (Björnberg et al. 1988; Förstner and Wittmann, 1981).

#### Organochlorines

**Fig. 7:**
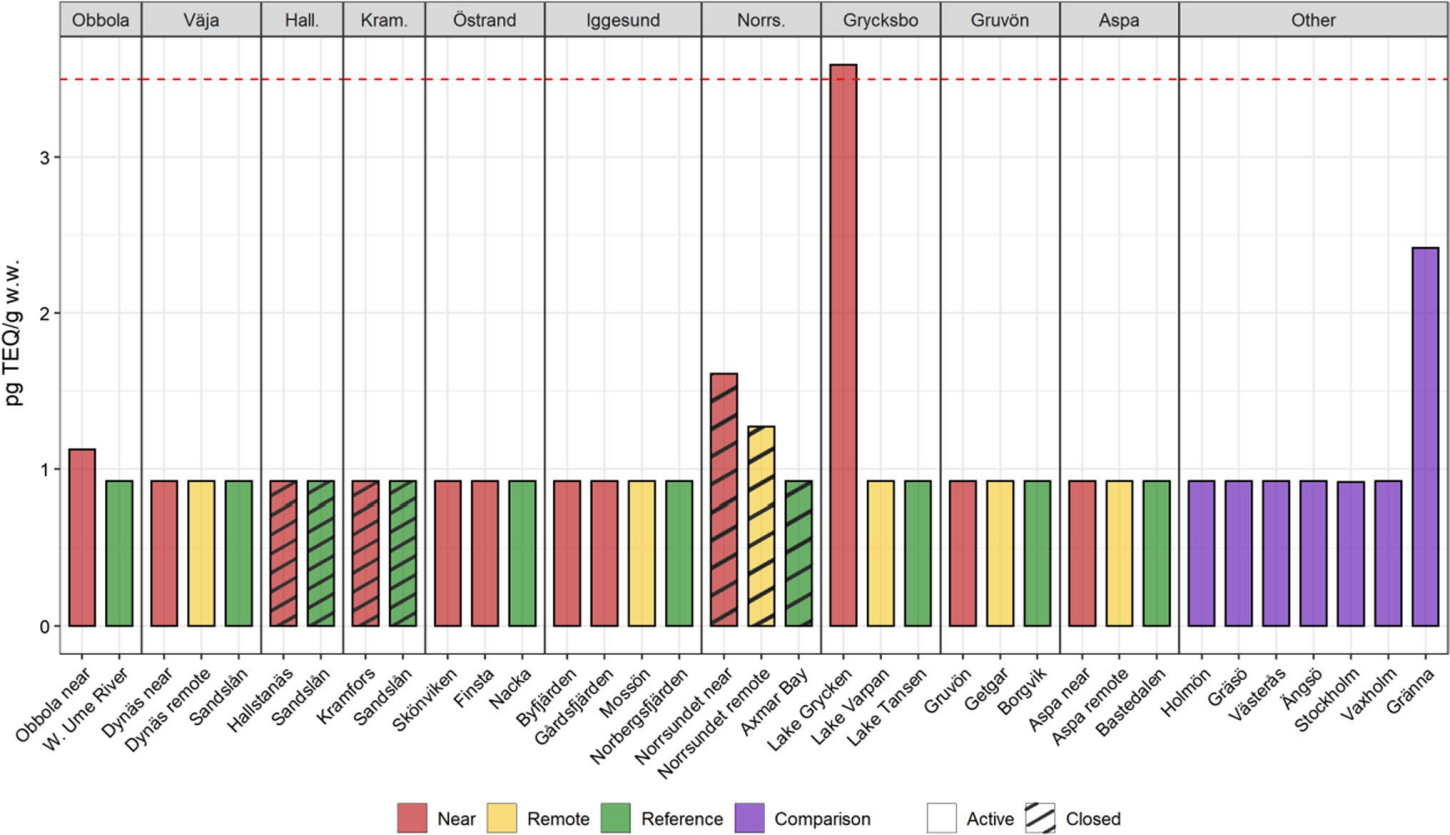
Lipid-normalized PCDD/F levels in perch from the study sites. Red line at 3.5 pg/g w.w. corresponds to marketing limit value within the European Union (EC 1881/2006)

In most cases, the PCDD/F levels were close to the detection limit of the analysis method (0.05–0.1 pg TEQ/g w.w., Fig. 7). This suggests, keeping in mind that a result below a relatively low detection limit also carries information, that it would be even more informative in the future, as reported in Dahmer et al. (2015), to measure PCDD/F levels in the liver that is a more fat-rich tissue. However, levels above detection limits were noted in a couple of cases, e.g., Lake Grycken. Underlying environmental factors like low bioproduction (Sandström et al. 2015) and, thereby, weak biodilution, limited sediment growth, and slow water turnover (Table 1) probably contributed. The theoretical exchange time of water in Lake Grycken is about 2 months compared to typically a few days in the coastal areas of the Baltic Sea (Bryhn et al. 2017). In a water area with slow water exchange, the effect of molecular diffusion from the sediments is more significant compared to an area with rapid water exchange when everything else is constant (Håkanson 1999).

**Fig. 8:**
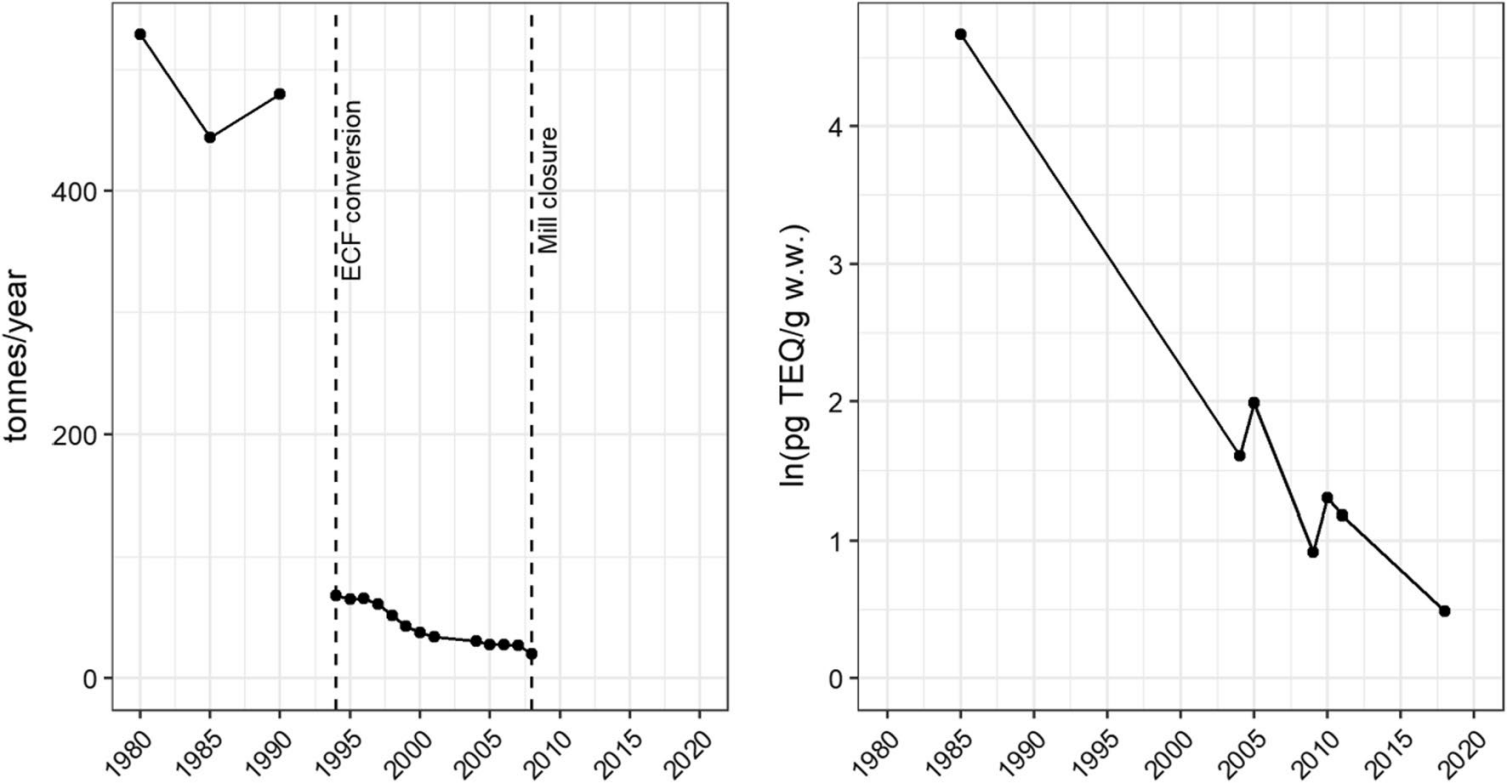
Left, time trends of AOX emissions from Norrsundet mill, data from Norrström and Karlsson (2015) (1980–1990), and environmental performance reporting from the mill (1994–2008). Right, PCDD/F levels in perch from the effluent receiving area. Data from Södergren 1989; Olsson et al. 2005; Karlsson and Malmaeus 2012 and actual survey

In the receiving area of the Norrsundet mill, there was a declining gradient with slightly elevated levels at the effluent discharge receiving sites. However, compared with historical data, the dioxin levels in perch have decreased (*p* < 0.05 for slope coefficient) (Fig. 8). The development of concentrations in fish follows relatively well the reduction of emissions of chlorinated substance (AOX), which can be linked to process changes and environmental protection measures taken. At the Norrsundet mill, the transition to ECF (Elemental Chlorine Free) bleaching occurred in 1994. In 2008, production at the Norrsundet mill ceased. It is yet possible to detect dioxin levels that are slightly higher than background premises, indicating that a specific, albeit small, bio-uptake occurs from the sediments either through molecular diffusion of dissolved dioxins into the organism or ingested via the food. Another possible contributing source of PCDD/Fs in the area was a sawmill that, until 1978, used chlorophenols to impregnate wood. The picture of declining PCDD/F levels in fish outside the Norrsundet mill is consistent with observations outside North American pulp mills (Pryke et al. 1995; Hagen et al. 1997; Pryke and Barden 2006; Dahmer et al. 2015). It is gratifying to note the apparent decline in dioxin levels. The last remnants of a vital cellulose industry environmental issue discussed for almost 50 years seem to be ending.

However, elevated levels of PCDD/Fs in pelagic fatty fish of the Baltic Sea, like herring (*Clupea harengus*) and salmon (*Salmo salar*), still is a severe environmental problem. Levels often exceed the EU marketing limits, and specific population groups (women of childbearing age and children) are advised by food safety authorities to limit their fish consumption. The results presented in this study show that temporal and spatial environmental monitoring can contribute to decision-making. Most pulp mills' receiving water areas in the coastal zone show low levels in non-migratory perch exposed to the legacy of the previous PCDD/F contamination. This indicates that measures against contaminated sediments outside pulp mills would be of minor importance for the remediation of the Baltic Sea's current dioxin problem. Instead, elevated levels in pelagic fish are primarily driven by atmospheric precipitation (Armitage et al. 2009; Assefa et al. 2019; SEPA 2021), similar to what has also been found in the North American Great Lakes (Pearson et al. 1998; Dahmer et al. 2015).

**Fig. 9:**
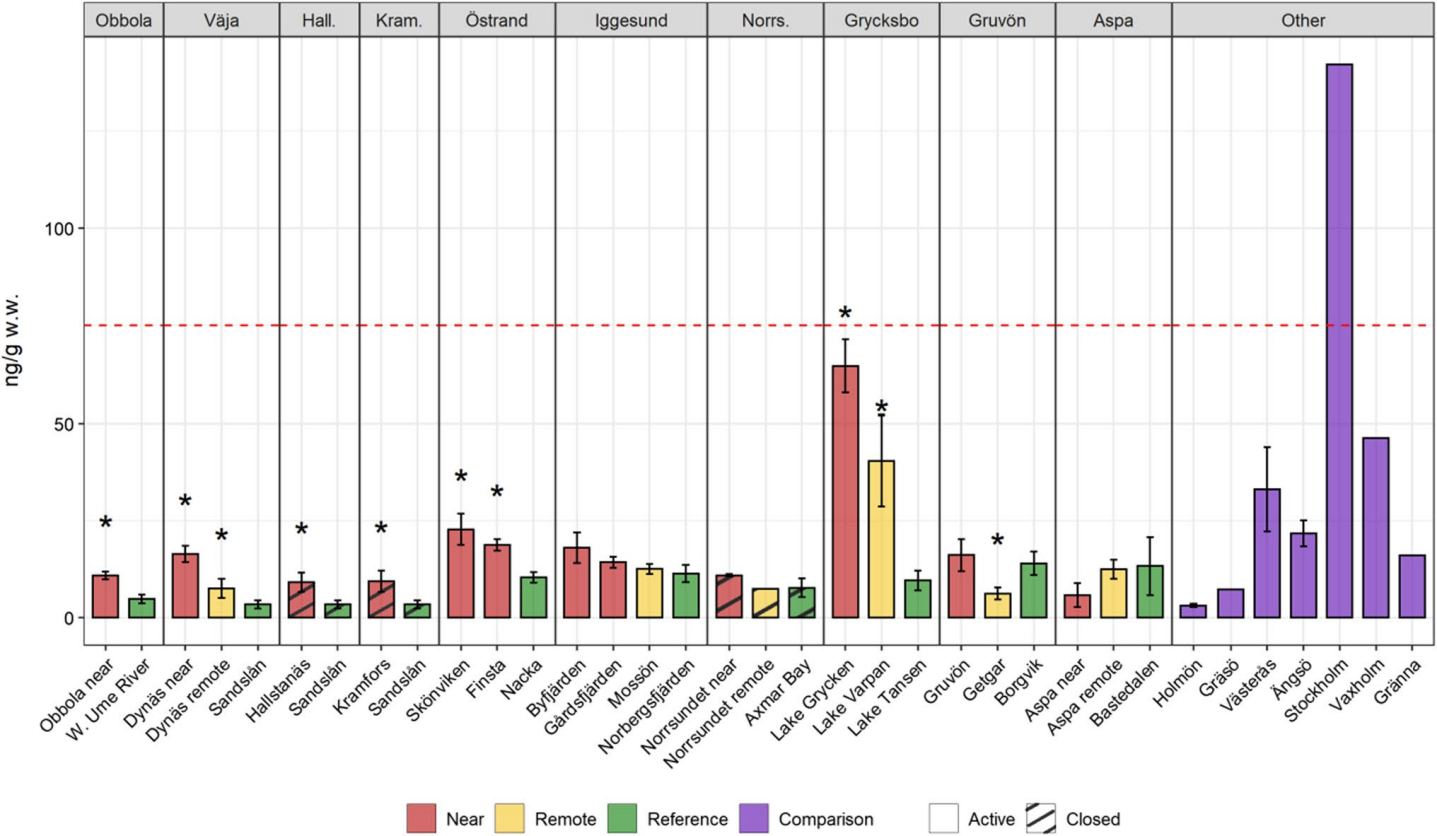
Lipid-normalized ∑PCB_6_ levels in perch from the study sites. Error bars show standard errors (SE). Stars indicate a significant difference toward reference (5% significance level). Red line at 75 ng/g w.w. corresponds to marketing limit value within the European Union (EC 1881/2006)

Significantly higher PCB levels in the near receiving areas were found at several mills (Obbola, Väja, Hallstanäs, Kramfors, Östrand, and Grycksbo, Fig. 9), probably reflecting the use of PCB oils in the infrastructure of the mills. A similar pattern as for Hg and PCDD/Fs, with the highest levels in the Grycksbo receiving water, was also noted for PCBs. The high levels in this area are likely due to the receiving water's characteristics, e.g., slow water turnover and low biodilution, rather than an exceptionally high load of PCBs. Except for Obbola and Östrand, most mills are situated near small municipalities (population < 5000). PCBs were widely used within society before it was banned in the 1970s. Not surprisingly, we found the highest PCB levels in the waters outside Stockholm, the largest city in Sweden (population approx. 1 million).

Dahlberg et al. (2020; 2021) have conducted detailed investigations of PCBs in one of our study areas, the receiving waters of the Väja mill. The fiber-rich sediments in this area contain moderately elevated PCB levels (~ 25 ng ∑PCB_7_/g d.w.). Measurements in benthic biota were interpreted as signs of bioaccumulation and biomagnification. Dahlberg et al. (2021) conclude that quantifying dispersal routes is essential for a proper risk assessment and risk management of contaminated sediments. The results presented in this study can be looked upon as an integrated quantification of the dispersal, showing (Fig. 9) that the spread and uptake of PCBs in the food web in the Väja area lead to slightly elevated levels of PCBs at the higher trophic level that predatory perch represents. Whether this is a risk that justifies measures or is a sign of an acceptable environmental situation for an area affected by industrial emissions for over a 100 years is not a scientific but a political question.

Lipid content normalized levels of all measured organochlorines are summarized in Appendix B. HCB and DDT generally showed weak or non-existing signs of bio-uptake in fish. However, in the receiving water of the Östrand mill, an elevated content of HCB (3 µg/kg w.w.) was measured, which can be linked to previous operations at a chlor-alkali plant. Compared to other studies from HCB-contaminated areas outside chlor-alkali plants, the levels were not remarkably high (Hinck et al. 2009; Huertas et al. 2016).

The DDT levels were elevated in the receiving area of the Grycksbo mill compared to other sites. However, the content was highest in the upstream reference lake Tansen (8 µg/kg w.w.). After investigation, it turned out that adjacent to this lake, a wool factory operated in the 1950s and 1960s, and blankets were impregnated with DDT. Thus, this is an excellent example of when fish can function as a sentinel to detect unknown hazards in the aquatic environment.

As discussed by Bignert et al. (2014), the relationship between chemical analytical error and other sources of variation, as well as the cost for collection, preparation of samples, and chemical analysis, will determine the number of individuals in each pool and the number of pools that should be analyzed to achieve high cost efficiency and good statistical power. When using pooled samples instead of analyzing individual fish, information at the individual level is, by definition, lost. This is not optimal since the underlying distribution and possible outliers are masked. Statistical comparisons of data from pooled samples also rely on randomly assigning individuals to a sample, a requirement that must not be overlooked. Despite these drawbacks, in our opinion, statistical comparison with acceptable precision that fulfill the criteria of an operative environmental monitoring program can be made.

### Health Status

#### Observations

**Fig. 10:**
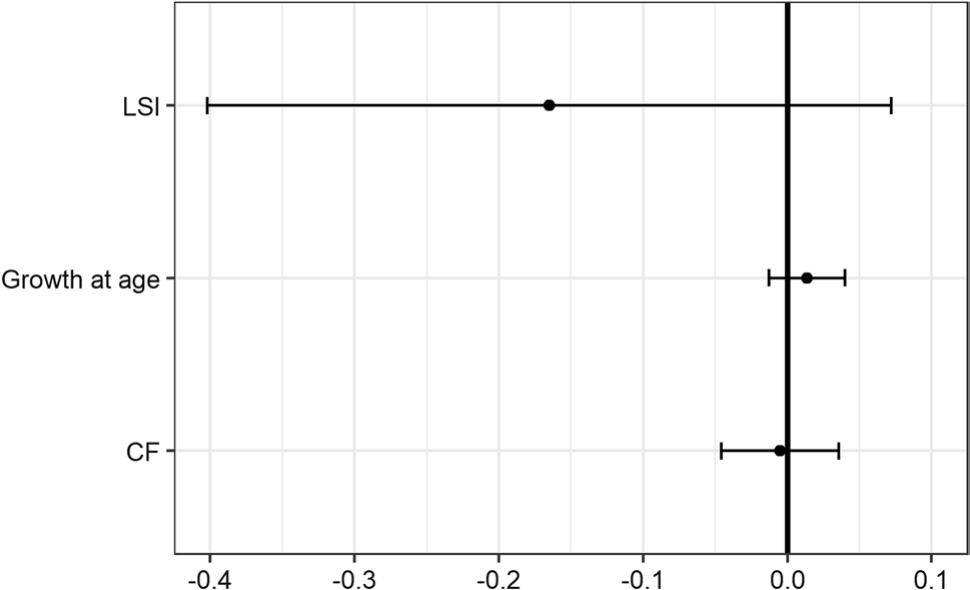
Results from a meta-analysis of CF, LSI, and growth at age in perch collected between 2017 and 2019, comparing near receiving sites with reference sites. Points mark estimated average responses, and intervals mark 95% confidence intervals for estimated averages. Intervals covering zero indicate no significant overall difference between perch from near receiving sites compared to perch from reference sites. GSI and SM were not considered due to low sample sizes for these indexes

The meta-analysis of the morphometric measures CF, LSI, and growth at age gave no indications of an overall response pattern (*p* > 0.05) (Fig. 10). Intervals covering zero indicate no overall response pattern, whereas intervals to the left of zero indicate a reduction in that index. Vice versa, intervals to the right indicate an increase. All intervals cover zero in this case. GSI and SM were excluded from the meta-analysis since too few sexually mature individuals were obtained at most sites. The only two mills regarded as having a sufficient sample size for evaluation of GSI and SM were Väja (all sites) and Grycksbo (Lake Grycken and Lake Tansen, not Lake Varpan). Future studies with larger sample sizes for GSI and SM will shed more light on the overall response pattern for these indexes. Summary data of morphological indexes are presented in Appendix C.

#### Evaluation

Outside Norrsundet and Iggesund, there are data records from earlier health surveys (Sandström et al. 2016). In the 1980s, several biomarkers (LSI, GSI, CF, specific blood parameters, skeletal deformations) indicated apparent health effects (Andersson et al. 1988; Sandström et al. 1988; Förlin et al. 1995). During the 1990s, the effect pattern was still clear but less pronounced (Sandström et al. 1997; Sandström and Neuman; 2003). From 2000 and onwards, decreasing but, in some cases, still, significant deviations in variables addressing reproduction and condition have been observed outside Norrsundet but not Iggesund (Sandström et al. 2016). During this time, several important protective measures were undertaken at the mills to reduce effluent toxicity (Norrström and Karlsson 2015). Process optimizations inside the mills, including improved stock washing, oxygen delignification, and handling of spill and condensate, have likely been the most important measures (Sandström et al. 2016). Clearly, the fish health has responded to the mitigative actions, but some deviations may persist. This is consistent with observations from Canada (Arciszewski et al. 2021; Ussery et al. 2021).

CF and LSI showed an effluent-associated increase in the Canadian EEM program (Lowell et al. 2003) but not in this study. In Canada, weight at age was generally increased, but growth at age (which is highly correlated with weight at age) showed no overall response pattern. The opposite, with a smaller LSI in the receiving areas, has also been observed and interpreted as a result of food limitation or residual habitat damage (Arciszewski et al. 2015). When comparing the statistically significant endpoints at each mill with the respective CES, significances exceeding CES were present only for LSI (Table 4). However, they were not in a direction that corresponds with the average response pattern for metabolic disruption in European perch noted by Sandström et al. (2005) nor white sucker (*Catostomus commersonii)* frequently used as sentinel species in Canadian surveys (McMaster et al. 2006; Ussery et al. 2021). For CF, no exceedances of CES were present. For growth at age, statistically significant differences (CES not derived) were found at Norrsundet, Gruvön, and Aspa, but only for the near receiving vs. reference, not the remote vs. reference. Outside the Norrsundet and Gruvön mills, perch had increased growth, while the opposite was found outside the Aspa mill (Table 4).

**Table 4:** Significant differences in biomarkers for health status. In bold indicates exceedance of CES. Plus sign indicates a higher value in near/remote receiving areas than reference sites; minus sign indicates lower in near/remote than in reference

	Significance (yes/no)									
Mill (mill status)	CF^1^	CF^2^	LSI^1^	LSI^2^	GSI^1^	GSI^2^	SM^1^	SM^2^	Growth^1^	Growth^2^
Obbola (active)	Yes −	^3^	Yes −	^3^	–	^3^	–	^3^	No	^3^
Väja (active)	Yes −	Yes–	**Yes–**	**Yes–**	No	No	Yes–	No	No	No
Östrand (active)	No	No	No	No	–	–	–	–	No	No
Iggesund (active)	No	No	Yes +	No	–	–	–	–	No	No
Norrsundet (closed)	Yes +	–	No	–	–	–	–	–	Yes +	–
Grycksbo (active)	No	Yes −	**Yes** −	**Yes** −	No	–	No	Yes −	No	No
Gruvön (active)	Yes +	Yes +	No	No	–	–	–	–	Yes +	No
Aspa (active)	No	No	No	No	–	–	–	–	Yes −	No

No CES is derived for sexual maturity (SM) and growth at age. “- “ not evaluated due to small sample size

^1^Near receiving vs. reference

^2^Remote vs. reference. ^3^no remote area at Obbola

In summary, CES was not exceeded at most mills for CF and LSI. The meta-analysis showed no indication of overall response patterns. The results point toward that Swedish paper and pulp mills generally do not negatively affect these endpoints, which is an improvement compared to historical conditions (Sandström et al. 2016).

As mentioned, for most mills, the sample sizes for the assessment of GSI and SM were unsatisfactory since few individuals had reached sexual maturity. To solve the problem of too few sexually mature individuals between 15 and 20 cm, it is suggested that fish of 20–25 cm are caught and included in the analysis as well. In this larger length span, a higher proportion of the individuals will be sexually mature, leading to increased sample sizes for comparisons of GSI. This addition to the methodology was successfully tested in the fall of 2020 outside a metal ore smelter in northern Sweden (Waldetoft et al. 2021).

Establishing good reference areas is essential to assess health conditions and reproductive capacity. In many cases, this is not trivial and needs to be carefully considered in the planning phase for future investigations. The assessments' reliability also increases if more than one reference area is established. Two main types of study designs for fish-health surveys occur, control-impact and gradient designs (Munkittrick et al. 2009). Choosing a reference area is a challenge for control-impact studies. Ideally, a reference site would be located upstream, in a similar habitat, free of confounding influences, with a natural barrier limiting movement between sites. Unfortunately, this situation is seldom fully achieved in coastal areas or large lakes. Therefore, it may be appropriate to initially work with two reference areas and study their inter-variability.

#### Statistical Considerations

To make a monitoring program cost-effective, it is necessary to limit the scope without compromising critical aspects of ecological field studies, e.g., the potential impacts of confounding factors, the ecological relevance of endpoints used, the influences of natural variability, concerns over statistical design issues, and possible genetic influences on species characteristics (Munkittrick 2009). One crucial parameter is the number of fish that need to be collected to distinguish any differences in health between areas with reasonable statistical certainty. For example, Munkittrick (1992) found insignificant improvement in white sucker variance and mean estimates of length and weight with a sample size above 16 individuals per site. Based on the data collected for this survey, a power analysis was conducted to find a sample size that yields sufficient power for the statistical tests. Calculations were focused on GSI since this variable is evaluated only for sexually mature individuals and thus acts as a bottleneck regarding sample sizes. Calculations were made for two cases based on GSI for sexually mature females in the Väja mill reference site and the Grycksbo mill reference site, the sites with the largest number of mature females, where 36 and 26 sexually mature females were caught, respectively (Table 2). From these sites, variance estimates were calculated. The required statistical power was set to 80%, and the numerical difference in mean GSI between a receiving water site and a reference site was set to 25% of GSI in the reference. The model was a one-way ANOVA with three groups: receiving water near, receiving water remote, and reference site. The remote receiving site mean GSI was set to the average of the reference and near receiving area. Results gave that between 16 and 31 sexually mature females are required. However, it should be mentioned that the standard approach of using a 5% significance level (risk of type I error) and a 20% chance of making a type II error (80% power) might not always be optimal in the case of impact assessment or environmental monitoring. It could be the case that making a Type II error is more costly than making a Type I error (Peterman and M'Gonigle 1992). In such cases, it could be an option to use statistical power higher than 80%.

Concerning the significance level used for assessing the health status of fish, the Canadian EEM approach and the approach presented in this study use a linear model (e.g., linear regression, ANOVA, or ANCOVA) to investigate site-specific differences. This ensures that the specified significance level is maintained throughout the analysis. An alternative, used in several Swedish surveys (Andersson et al. 1988; Sandström and Neuman 2003), is to use separate *t*-tests between sites, genders, and length classes. However, this leads to many *t*-tests being performed, each with *α* = 0.05. The consequence is that the overall *α* is larger than 0.05, meaning an increased risk of falsely rejecting the null hypothesis in favor of the alternative hypothesis and, thus, the risk of drawing a false conclusion. Therefore, using a linear model followed by a suitable post hoc test is preferable from a statistical standpoint.

## Conclusions and Prospects

The overall picture is that the levels of examined pollutants, with some exceptions, were not noticeably higher in the receiving waters investigated, neither in relation to nearby reference areas nor comparison areas from Swedish inland and coastal waters. Based on comparisons with historical data, the trend regarding levels of contaminants in fish in the cellulose industry's receiving waters is generally decreasing. Regarding fish health, with reservation for the reproductive variables where the sample sizes in most cases were too small, the overall picture is that fish health is not impaired in the receiving waters compared to the reference areas.

After the modifications discussed,

The method tested in the project should have the potential to become a relevant and cost-effective part of industries' ongoing environmental monitoring to have a follow-up and control over historical emissions to sediments over time. Studies of the kind carried out may also improve ecological understanding and guide decision-makers on possible remedial measures connected with contaminated sediments. In cases where it is judged that natural recovery is appropriate, i.e., where no physical measures are performed to remove or limit the impact from sediment contaminants, fish surveys should be an effective way of monitoring whether the recovery follows the expected course.

The case study areas have been receiving waters outside cellulose industries. The methodology has also been successfully tested outside a metal smelter (Waldetoft et al. 2021). It can likely be applied generally in aquatic ecosystems where a historical load of metals and persistent organic compounds have contaminated the sediments. The part of the monitoring program that pertains to fish health is not limited to areas with sediment pollution but could also be used to assess ongoing emissions outside industries, municipal treatment plants, or other point sources. The presented survey strategy is similar to the Canadian EEM program, successfully applied nationally to evaluate the cellulose and mining industries. We consider that this should be the way forward also in the Swedish environmental monitoring programs outside industries and look forward to making more international comparisons between results in the future, expanding the overall knowledge about the impact of contaminants on aquatic life.

## Appendix A: Levels of Metals

See Tables

**Table 5:** Levels of Cr, Ni, Cu, Zn, As, Cd, Pb (mg/kg d.w.) in *Perca fluviatilis* of length 15–20 cm. mean ± sd

Mill	Site	Cr	Ni	Cu	Zn	As	Cd	Pb	n^a^
Obbola	Obbola near	0.0217 ± 0.000441	0.585 ± 0.449	7.94 ± 0.698	121 ± 6.56	3.07 ± 1.57	1.6 ± 0.576	0.0594 ± 0.0355	6
Obbola	W. Ume River	0.021 ± 0.000424	4.8 ± 7.07	11.2 ± 1.57	108 ± 3.62	6.02 ± 0.376	0.917 ± 0.709	0.0533 ± 0.00978	3
Väja	Dynäs near	0.0617 ± 0.0153	0.0827 ± 0.00551	13.7 ± 2.08	120 ± 0	3.03 ± 1.07	1.36 ± 0.67	0.033 ± 0.01	3
Väja	Dynäs remote	0.0913 ± 0.0423	0.156 ± 0.0867	9.97 ± 1.05	117 ± 5.77	2.17 ± 1.74	1.64 ± 0.87	0.03 ± 0.004	3
Väja	Hallstanäs	0.063 ± 0.0131	0.09 ± 0.0624	9.93 ± 3.1	116 ± 16.9	3.5 ± 1.82	1.04 ± 0.0981	0.00833 ± 0.0118	3
Väja	Kramfors	0.102 ± 0.111	0.098 ± 0.102	12.5 ± 2.12	115 ± 7.07	3.75 ± 0.212	1.46 ± 1.05	0.02 ± 0.0099	2
Väja	Sandslån	0.123 ± 0.128	0.0767 ± 0.0981	6.1 ± 0.5	102 ± 7.64	0.99 ± 0.184	0.65 ± 0.262	0.00383 ± 0.00101	3
Östrand	Skönviken	0.01 ± 0	0.236 ± 0.502	7.08 ± 1.2	85.2 ± 10.4	3.35 ± 1.03	0.141 ± 0.0481	0.0173 ± 0.0265	10^b^
Östrand	Finsta	0.0252 ± 0.0333	0.408 ± 0.568	6.39 ± 0.942	73.6 ± 12.4	5.29 ± 1.75	0.185 ± 0.132	0.00777 ± 0.00962	10^b^
Östrand	Nacka	0.01 ± 0	0.035 ± 0	5.55 ± 1.41	62.2 ± 9.58	4.31 ± 0.679	0.127 ± 0.051	0.00292 ± 0.0029	10^b^
Iggesund	Byfjärden	0.0251 ± 0.0261	0.035 ± 0	6.09 ± 1.04	73.5 ± 13.2	2.11 ± 2.11	0.193 ± 0.0476	0.0362 ± 0.0363	3
Iggesund	Gårdsfjärden	0.01 ± 0	0.035 ± 0	5.82 ± 1.21	71.3 ± 18.5	3.95 ± 0.628	0.444 ± 0.225	0.0155 ± 0.0234	3
Iggesund	Mössön	0.01 ± 0	0.035 ± 0	9.43 ± 1.06	89 ± 5.76	5.84 ± 0.525	0.382 ± 0.00413	0.002 ± 0	3
Iggesund	Norbergsfjärden	0.0273 ± 0.0299	0.035 ± 0	7.03 ± 1.47	73.4 ± 19.9	4.4 ± 1.66	0.55 ± 0.289	0.002 ± 0	3
Norrsundet	Norrsundet near	0.0194 ± 5.65e-05	0.0777 ± 0.000226	8.68 ± 0.43	94.5 ± 6.09	3.54 ± 0.65	0.142 ± 0.00263	0.00583 ± 1.69e-05	3
Norrsundet	Norrsundet remote	0.0201 ± NA	0.0802 ± NA	7.62 ± NA	96.3 ± NA	3.85 ± NA	0.152 ± NA	0.00602 ± NA	1
Norrsundet	Axmar Bay	0.0202 ± 0.000787	0.0806 ± 0.00315	7.42 ± 1.42	98.4 ± 15.9	5.24 ± 0.886	0.153 ± 0.0464	0.00605 ± 0.000236	3
Grycksbo	Lake Grycken	0.189 ± 0.174	0.0487 ± 0.0301	8.6 ± 1.05	140 ± 17.3	0.447 ± 0.139	3.13 ± 0.907	0.131 ± 0.0908	3
Grycksbo	Lake Varpan	0.115 ± 0.152	0.048 ± 0.0271	17.7 ± 3.51	143 ± 5.77	0.723 ± 0.0929	6.9 ± 0.346	0.094 ± 0.0487	3
Grycksbo	Lake Tansen	0.194 ± 0.198	0.038 ± 0.0156	6.93 ± 0.513	123 ± 5.77	0.29 ± 0.0265	3.7 ± 0.265	0.096 ± 0.0826	3
Gruvön	Gruvön	0.0324 ± 0.0169	0.0891 ± 0.00442	8.77 ± 0.605	114 ± 2.42	1.37 ± 0.544	2.41 ± 0.654	0.0297 ± 0.0227	3
Gruvön	Getgar	0.0203 ± 0.000217	0.0813 ± 0.000869	9.34 ± 0.97	112 ± 1.87	1.49 ± 0.383	1.23 ± 0.262	0.026 ± 0.0081	3
Gruvön	Borgvik	0.0749 ± 0.0642	0.0872 ± 0.00215	8.13 ± 1.14	110 ± 2.4	0.709 ± 0.191	2.6 ± 0.489	0.0294 ± 0.00686	3
Aspa	Aspa near	0.052 ± 0.0251	0.047 ± 0.0298	9.9 ± 2.15	127 ± 5.77	1.01 ± 0.338	2.03 ± 0.404	0.06 ± 0.0223	3
Aspa	Aspa remote	0.0733 ± 0.0398	0.107 ± 0.15	8.47 ± 0.737	123 ± 5.77	1.1 ± 0.365	1.73 ± 0.404	0.0413 ± 0.00379	3
Aspa	Bastedalen	0.075 ± 0.0355	0.0313 ± 0.0196	9.03 ± 0.379	123 ± 11.5	1.33 ± 0.208	3.17 ± 0.907	0.207 ± 0.0874	3
Comparison site	Holmön	0.0202 ± 0.00102	0.337 ± 0.447	8.08 ± 1.13	98.4 ± 15	4.58 ± 0.471	0.245 ± 0.0131	0.00605 ± 0.000306	3
Comparison site	Västerås	0.0213 ± 0.000315	0.0853 ± 0.00126	9.09 ± 1.39	119 ± 1.77	3.75 ± 0.782	0.569 ± 0.0536	0.0064 ± 9.46e-05	3
Comparison site	Ängsö	0.0215 ± 0.000813	0.0861 ± 0.00325	9.58 ± 4.65	112 ± 11	3.98 ± 1.57	0.657 ± 0.228	0.00646 ± 0.000244	3
Comparison site	Gränna	0.02 ± NA	0.08 ± NA	7.4 ± NA	110 ± NA	2.2 ± NA	1.7 ± NA	0.014 ± NA	1

a = pooled samples of equal amount of tissue from 4 to 10 individuals. b = individual samples

5 and

**Table 6:** Hg levels, total and weight normalized (mg/kg w.w.) in *Perca fluviatilis* of length 15–20 cm. Mean ± sd

Mill	Site	Hg_tot_	Total weight (kg)	Hg_norm_	n^a^
Obbola	Obbola near	0.0833 ± 0.0163	0.0586 ± 0.00288	0.219 ± 0.0449	6
Obbola	W. Ume River	0.05 ± 0.00529	0.0665 ± 0.00321	0.124 ± 0.0111	3
Väja	Dynäs near	0.082 ± 0.0164	0.0587 ± 0.000577	0.215 ± 0.0426	3
Väja	Dynäs remote	0.125 ± 0.0522	0.0557 ± 0.00586	0.333 ± 0.131	3
Hallstanäs	Hallstanäs	0.233 ± 0.0862	0.0553 ± 0.00306	0.623 ± 0.218	3
Kramfors	Kramfors	0.157 ± 0.0115	0.0633 ± 0.00306	0.397 ± 0.0379	3
Väja	Sandslån	0.193 ± 0.0208	0.064 ± 0.002	0.487 ± 0.0548	3
Östrand	Skönviken	0.0995 ± 0.0349	0.0638 ± 0.0162	0.251 ± 0.0757	10^b^
Östrand	Finsta	0.0944 ± 0.0466	0.0815 ± 0.018	0.212 ± 0.0914	10^b^
Östrand	Nacka	0.0671 ± 0.0124	0.0561 ± 0.0121	0.179 ± 0.0264	10^b^
Iggesund	Byfjärden	0.0744 ± 0.0385	0.0794 ± 0.00349	0.169 ± 0.0846	3
Iggesund	Gårdsfjärden	0.116 ± 0.0674	0.0802 ± 0.0149	0.267 ± 0.169	3
Iggesund	Mössön	0.0686 ± 0.023	0.0742 ± 0.00979	0.16 ± 0.0476	3
Iggesund	Norbergsfjärden	0.0599 ± 0.0222	0.0705 ± 0.00569	0.143 ± 0.0485	3
Norrsundet	Norrsundet near	0.14 ± 0.01	0.0916 ± 0.0038	0.299 ± 0.0232	3
Norrsundet	Norrsundet remote	0.074 ± NA	0.0682 ± NA	0.181 ± NA	1
Norrsundet	Axmar Bay	0.0553 ± 0.0176	0.0731 ± 0.0172	0.129 ± 0.0282	3
Grycksbo	Lake Grycken	0.37 ± 0.01	0.0529 ± 0.00288	1.01 ± 0.0468	3
Grycksbo	Lake Varpan	0.093 ± 0.017	0.0528 ± 0.00295	0.255 ± 0.0519	3
Grycksbo	Lake Tansen	0.197 ± 0.0208	0.0587 ± 0.000656	0.515 ± 0.0562	3
Gruvön	Gruvön	0.123 ± 0.0656	0.073 ± 0.00854	0.287 ± 0.133	5
Gruvön	Getgar	0.0706 ± 0.0132	0.063 ± 0.00769	0.179 ± 0.0348	5
Gruvön	Borgvik	0.158 ± 0.0217	0.0611 ± 0.00454	0.407 ± 0.061	5
Aspa	Aspa near	0.0353 ± 0.00404	0.0599 ± 0.00357	0.0915 ± 0.00813	3
Aspa	Aspa remote	0.0403 ± 0.00513	0.0628 ± 0.0018	0.102 ± 0.0141	3
Aspa	Bastedalen	0.0383 ± 0.0129	0.0648 ± 0.0024	0.0958 ± 0.0311	3
Comparison site	Holmön	0.0627 ± 0.0197	0.0973 ± 0.0132	0.129 ± 0.0368	3
Comparison site	Gräsö	0.0803 ± NA	0.035 ± NA	0.26 ± NA	1
Comparison site	Västerås	0.0737 ± 0.00814	0.0804 ± 0.0126	0.167 ± 0.00633	3
Comparison site	Ängsö	0.072 ± 0.0161	0.064 ± 0.00838	0.18 ± 0.0299	3
Comparison site	Stockholm	0.141 ± NA	0.038 ± NA	0.442 ± NA	1
Comparison site	Vaxholm	0.101 ± NA	0.045 ± NA	0.296 ± NA	1
Comparison site	Gränna	0.087 ± NA	0.144 ± NA	0.148 ± NA	1

a = pooled samples of equal amount of tissue from 4 to 10 individuals. b = individual samples

6

## Appendix B: Levels of Organochlorines

See Tables

**Table 7:** Levels of PCB, HCB and DDT (ng/g w.w.) in *Perca fluviatilis* of length 15–20 cm. Mean ± sd

Mill	Site	PCB 28	PCB 52	PCB 101	PCB 138	PCB 153	PCB 180	HCB	pp-DDE	pp-DDD	pp-DDT	n^a^
Obbola	Obbola near	0.02 ± 0	0.025 ± 0	0.209 ± 0.0206	0.607 ± 0.0678	0.764 ± 0.0885	0.192 ± 0.0283	0.0603 ± 0.00526	0.397 ± 0.118	0.0353 ± 0.00996	0.0188 ± 0.0174	6
Obbola	W. Ume River	0.02 ± 0	0.025 ± 0	0.0851 ± 0.0212	0.286 ± 0.0605	0.387 ± 0.0617	0.0963 ± 0.0176	0.0694 ± 0.00634	0.209 ± 0.0404	0.00633 ± 0.011	0.0165 ± 0.0143	3
Väja	Dynäs near	0.02 ± 0	0.025 ± 0	0.304 ± 0.0399	0.886 ± 0.118	1.01 ± 0.111	0.395 ± 0.0373	0.0506 ± 0.00937	0.298 ± 0.0454	0.069 ± 0.0241	0.0527 ± 0.00755	3
Väja	Dynäs remote	0.02 ± 0	0.025 ± 0	0.193 ± 0.0618	0.477 ± 0.136	0.542 ± 0.127	0.147 ± 0.0329	0.0546 ± 0.007	0.302 ± 0.0343	0.0889 ± 0.0341	0.0552 ± 0.00982	3
Väja	Hallstanäs	0.02 ± 0	0.025 ± 0	0.203 ± 0.0312	0.519 ± 0.117	0.596 ± 0.156	0.198 ± 0.0541	0.0509 ± 0.00617	0.293 ± 0.0222	0.0774 ± 0.0107	0.0493 ± 0.0165	3
Väja	Kramfors	0.02 ± 0	0.0217 ± 0.00289	0.203 ± 0.0384	0.573 ± 0.14	0.649 ± 0.143	0.217 ± 0.0682	0.0683 ± 0.0102	0.365 ± 0.0705	0.215 ± 0.191	0.0732 ± 0.0116	3
Väja	Sandslån	0.02 ± 0	0.025 ± 0	0.0876 ± 0.0149	0.214 ± 0.053	0.234 ± 0.0606	0.0637 ± 0.017	0.0396 ± 0.00208	0.14 ± 0.0339	0.036 ± 0.00365	0.028 ± 0.0224	3
Östrand	Skönviken	0.038 ± 0.0156	0.124 ± 0.0272	0.656 ± 0.0926	1.28 ± 0.179	1.36 ± 0.175	0.558 ± 0.0926	0.446 ± 0.0686	0.237 ± 0.0257	0.0518 ± 0.00691	0.0779 ± 0.00563	3
Östrand	Finsta	0.06 ± 0.0119	0.0353 ± 0.0179	0.37 ± 0.0411	0.98 ± 0.0588	1.03 ± 0.0654	0.479 ± 0.0381	0.277 ± 0.0169	0.312 ± 0.0296	0.068 ± 0.00989	0.121 ± 0.0235	3
Östrand	Nacka	0.0233 ± 0.0058	0.0283 ± 0.00577	0.226 ± 0.0218	0.606 ± 0.0629	0.655 ± 0.0788	0.26 ± 0.0386	0.153 ± 0.0277	0.211 ± 0.0373	0.028 ± 0.00724	0.0564 ± 0.011	3
Iggesund	Byfjärden	0.057 ± 0.0165	0.11 ± 0.0324	0.414 ± 0.0909	0.773 ± 0.157	0.889 ± 0.173	0.361 ± 0.0938	0.186 ± 0.0717	0.348 ± 0.0491	0.0812 ± 0.016	0.0159 ± 0.0103	3
Iggesund	Gårdsfjärden	0.056 ± 0.0077	0.0864 ± 0.0155	0.297 ± 0.0311	0.681 ± 0.0625	0.797 ± 0.0715	0.301 ± 0.0368	0.198 ± 0.0174	0.485 ± 0.0167	0.128 ± 0.0322	0.1 ± 0.0239	3
Iggesund	Mössön	0.0217 ± 0.00289	0.0267 ± 0.00289	0.236 ± 0.0156	0.646 ± 0.0656	0.767 ± 0.072	0.297 ± 0.0299	0.194 ± 0.0199	0.5 ± 0.0416	0.0756 ± 0.00632	0.0947 ± 0.00938	3
Iggesund	Norbergs-fjärden	0.0217 ± 0.00289	0.0267 ± 0.00289	0.196 ± 0.0409	0.568 ± 0.114	0.698 ± 0.127	0.26 ± 0.0417	0.152 ± 0.0511	0.44 ± 0.115	0.0377 ± 0.00945	0.0566 ± 0.00633	3
Norrsundet	Norrsundet near	0.02 ± 0	0.025 ± 0	0.153 ± 0.116	0.647 ± 0.0355	0.735 ± 0.0447	0.226 ± 0.00663	0.0689 ± 0.00777	0.471 ± 0.0799	0.12 ± 0.00563	0.0272 ± 0.00778	3
Norrsundet	Norrsundet remote	0.02 ± NA	0.025 ± NA	0.144 ± NA	0.426 ± NA	0.497 ± NA	0.134 ± NA	0.0587 ± NA	0.172 ± NA	0 ± NA	0 ± NA	1
Norr-sundet	Axmar Bay	0.0555 ± 0.0615	0.025 ± 0	0.114 ± 0.0425	0.368 ± 0.107	0.423 ± 0.129	0.126 ± 0.0374	0.0708 ± 0.00454	0.16 ± 0.03	0.0335 ± 0.0346	0.015 ± 0.0136	3
Grycksbo	Lake Grycken	0.02 ± 0	0.165 ± 0.0395	1.32 ± 0.292	3.38 ± 0.183	4.05 ± 0.408	1.82 ± 0.119	0.129 ± 0.0279	0.489 ± 0.0197	0.686 ± 0.102	0.009 ± 0	3
Grycksbo	Lake Varpan	0.02 ± 0	0.025 ± 0	0.493 ± 0.113	2.17 ± 0.539	2.76 ± 0.671	1.21 ± 0.352	0.0297 ± 0.00289	0.603 ± 0.121	0.137 ± 0.018	0.009 ± 0	3
Grycksbo	Lake Tansen	0.02 ± 0	0.0233 ± 0.00289	0.156 ± 0.0171	0.584 ± 0.122	0.738 ± 0.153	0.28 ± 0.0687	0.0347 ± 0.00153	1.26 ± 0.155	0.149 ± 0.0162	0.009 ± 0	3
Gruvön	Gruvön	0.02 ± 0	0.046 ± 0.019	0.333 ± 0.0877	0.822 ± 0.199	0.904 ± 0.227	0.283 ± 0.0502	0.0675 ± 0.0106	0.355 ± 0.0656	0.0731 ± 0.00795	0.0326 ± 0.00386	3
Gruvön	Getgar	0.02 ± 0	0.025 ± 0	0.111 ± 0.0394	0.357 ± 0.0773	0.403 ± 0.079	0.125 ± 0.0322	0.0346 ± 0.00273	0.321 ± 0.0827	0.0546 ± 0.00739	0.0239 ± 0.00336	3
Gruvön	Borgvik	0.02 ± 0	0.025 ± 0	0.18 ± 0.0327	0.838 ± 0.151	1.01 ± 0.187	0.361 ± 0.0702	0.031 ± 0.00436	0.487 ± 0.0875	0.0377 ± 0.00968	0.0076 ± 0.0132	3
Aspa	Aspa near	0.02 ± 0	0.025 ± 0	0.0904 ± 0.0282	0.418 ± 0.153	0.486 ± 0.184	0.166 ± 0.0755	0.104 ± 0.00284	0.775 ± 0.248	0.0317 ± 0.0166	0.0495 ± 0.0248	3
Aspa	Aspa remote	0.02 ± 0	0.025 ± 0	0.157 ± 0.0293	0.763 ± 0.137	0.879 ± 0.131	0.308 ± 0.0513	0.14 ± 0.0811	1.21 ± 0.251	0.0788 ± 0.0153	0.0501 ± 0.0283	3
Aspa	Bastedalen	0.02 ± 0	0.025 ± 0	0.18 ± 0.0982	0.771 ± 0.381	0.919 ± 0.437	0.357 ± 0.166	0.0371 ± 0.0019	1.21 ± 0.727	0.0873 ± 0.026	0.0793 ± 0.0615	3
Comparison site	Holmön	0.02 ± 0	0.025 ± 0	0.0406 ± 0.0186	0.181 ± 0.0153	0.247 ± 0.0272	0.0669 ± 0.0132	0.0874 ± 0.00431	0.175 ± 0.0122	0 ± 0	0 ± 0	3
Comparison site	Gräsö	0.0155 ± NA	0.0245 ± NA	0.095 ± NA	0.39 ± NA	0.54 ± NA	0.25 ± NA	NaN ± NA	NaN ± NA	NaN ± NA	NaN ± NA	1
Comparison site	Västerås	0.02 ± 0	0.131 ± 0.0278	0.837 ± 0.204	2.22 ± 0.542	2.27 ± 0.617	0.721 ± 0.155	0.0372 ± 0.00244	1.3 ± 0.302	0.156 ± 0.0244	0.0564 ± 0.00676	3
Comparison site	Ängsö	0.02 ± 0	0.025 ± 0	0.332 ± 0.0389	1.25 ± 0.167	1.39 ± 0.213	0.387 ± 0.065	0.0381 ± 0.00489	1.2 ± 0.156	0.0977 ± 0.00882	0.0736 ± 0.0046	3
Comparison site	Stockholm	0.92 ± NA	1.8 ± NA	6.1 ± NA	4.9 ± NA	6.4 ± NA	2.9 ± NA	NaN ± NA	NaN ± NA	NaN ± NA	NaN ± NA	1
Comparison site	Vaxholm	0.125 ± NA	0.35 ± NA	1.6 ± NA	1.7 ± NA	3 ± NA	0.83 ± NA	NaN ± NA	NaN ± NA	NaN ± NA	NaN ± NA	1
Comparison site	Gränna	0.02 ± NA	0.025 ± NA	0.186 ± NA	0.99 ± NA	1.08 ± NA	0.338 ± NA	0.107 ± NA	1.62 ± NA	0.367 ± NA	0.052 ± NA	1

a = pooled samples of equal amount of tissue from 4 to 10 individuals

7,

**Table 8:** PCDD levels (pg/g w.w.) in *Perca fluviatilis* of length 15–20 cm

Mill	Site	2,3,7,8-TetraCDD	1,2,3,7,8-PentaCDD	1,2,3,4,7,8-HexaCDD	1,2,3,6,7,8-HexaCDD	1,2,3,7,8,9-HexaCDD	1,2,3,4,6,7,8- HeptaCDD	OktaCDD	n^a^
Obbola	Obbola near	< 0.13	< 0.19	< 0.25	< 0.25	< 0.25	< 0.3	< 0.34	1
Obbola	W. Ume River	< 0.093	< 0.21	< 0.26	< 0.26	< 0.26	< 0.38	< 0.42	1
Väja	Väja near	< 0.18	< 0.27	< 0.32	< 0.32	< 0.32	< 0.3	< 0.46	1
Väja	Väja remote	< 0.13	< 0.21	< 0.39	< 0.39	< 0.39	< 0.37	< 0.48	1
Väja	Sandslån	< 0.15	< 0.22	< 0.31	< 0.31	< 0.31	< 0.48	< 0.76	1
Väja	Hallstanäs	< 0.14	< 0.28	< 0.43	< 0.43	< 0.43	< 0.67	< 0.8	1
Väja	Kramfors	< 0.13	< 0.2	< 0.34	< 0.34	< 0.34	< 0.47	< 0.79	1
Östrand	Skönviken	< 0.11	< 0.091	< 0.19	< 0.19	< 0.19	< 0.26	< 0.34	1
Östrand	Finsta	< 0.11	< 0.11	< 0.25	< 0.25	< 0.25	< 0.31	< 0.53	1
Östrand	Nacka	< 0.11	< 0.1	< 0.21	< 0.21	< 0.21	< 0.28	< 0.43	1
Iggesund	Byfjärden	< 0.12	< 0.13	< 0.18	< 0.18	< 0.18	< 0.39	< 0.46	1
Iggesund	Gårdsfjärden	< 0.1	< 0.13	< 0.23	< 0.23	< 0.23	< 0.26	< 0.41	1
Iggesund	Mössön	< 0.13	< 0.13	< 0.25	< 0.25	< 0.25	< 0.29	< 0.43	1
Iggesund	Norbergsfjärden	< 0.11	< 0.12	< 0.15	< 0.15	< 0.15	< 0.16	< 0.22	1
Norrsundet	Norrsundet near	< 0.2	< 0.15	< 0.43	< 0.43	< 0.43	< 0.43	< 0.66	1
Norrsundet	Norrsundet remote	< 0.087	< 0.16	< 0.25	< 0.25	< 0.25	< 0.42	< 0.61	1
Norrsundet	Axmar Bay	< 0.16	< 0.24	< 0.25	< 0.25	< 0.25	< 0.4	< 0.54	1
Grycksbo	Lake Grycken	< 0.047	< 0.05	< 0.067	< 0.067	< 0.067	< 0.057	4,1	1
Grycksbo	Lake Varpan	< 0.084	< 0.089	< 0.2	< 0.2	< 0.2	< 0.091	< 2.5	1
Grycksbo	Lake Tansen	< 0.075	< 0.094	< 0.15	< 0.15	< 0.15	< 0.17	< 0.18	1
Gruvön	Gruvön	< 0.077	< 0.11	< 0.19	< 0.19	< 0.19	< 0.24	< 3.2	1
Gruvön	Getgar	< 0.14	< 0.21	< 0.28	< 0.28	< 0.28	< 0.44	< 0.73	1
Gruvön	Borgvik	< 0.17	< 0.27	< 0.31	< 0.31	< 0.31	< 0.47	< 2.6	1
Aspa	Aspa near	< 0.11	< 0.19	< 0.66	< 0.66	< 0.66	< 0.75	< 0.98	1
Aspa	Aspa remote	< 0.1	< 0.17	< 0.57	< 0.57	< 0.57	< 0.98	< 1.5	1
Aspa	Bastedalen	< 0.12	< 0.16	< 0.5	< 0.5	< 0.5	< 0.64	< 0.88	1
Comparison site	Holmön	< 0.095	< 0.17	< 0.25	< 0.25	< 0.25	< 0.4	< 0.47	1
Comparison site	Västerås	< 0.12	< 0.21	< 0.23	< 0.23	< 0.23	< 0.47	< 1.4	1
Comparison site	Ängsö	< 0.12	< 0.24	< 0.28	< 0.28	< 0.28	< 0.61	< 4	1
Comparison site	Stockholm	< 0.07	< 0.096	< 0.1	< 0.1	< 0.1	< 0.2	< 0.27	1
Comparison site	Vaxholm	< 0.072	< 0.098	< 0.1	< 0.1	< 0.1	< 0.15	< 0.16	1
Comparison site	Gränna	< 0.34	< 0.41	< 0.61	< 0.61	< 0.61	< 0.52	< 0.56	1
Comparison site	Gräsö	< 0.04	< 0.077	< 0.26	< 0.26	< 0.26	< 0.21	< 0.25	1

a = pooled samples of equal amount of tissue from 4 to 10 individuals

8,

**Table 9:** PCDF levels (pg/g w.w.) in *Perca fluviatilis* of length 15–20 cm

Mill	Sample	2,3,7,8-Tetra CDF	1,2,3,7, 8-Penta CDF	2,3,4,7, 8-Penta CDF	1,2,3,4,7, 8-Hexa CDF	1,2,3,6, 7, 8-Hexa CDF	1,2,3,7, 8,9-Hexa CDF	2,3,4,6, 7,8-Hexa CDF	1,2,3,4,6, 7,8-Hepta CDF	1,2,3,4,7, 8,9-Hepta CDF	Okta CDF	n^a^
	Obbola near	< 0.19	< 0.25	< 0.25	< 0.22	< 0.22	< 0.22	< 0.22	< 0.27	< 0.27	< 0.31	1
Obbola	W. Ume River	0.46	< 0.23	< 0.23	< 0.29	< 0.29	< 0.29	< 0.29	< 0.22	< 0.22	< 0.38	1
Väja	Väja near	< 0.2	< 0.22	< 0.22	< 0.25	< 0.25	< 0.25	< 0.25	< 0.2	< 0.2	< 0.41	1
Väja	Väja remote	< 0.22	< 0.24	< 0.24	< 0.28	< 0.28	< 0.28	< 0.28	< 0.23	< 0.23	< 0.43	1
Väja	Sandslån	< 0.25	< 0.25	< 0.25	< 0.36	< 0.36	< 0.36	< 0.36	< 0.32	< 0.32	< 0.68	1
Väja	Hallstanäs	< 0.31	< 0.2	< 0.2	< 0.24	< 0.24	< 0.24	< 0.24	< 0.36	< 0.36	< 0.72	1
Väja	Kramfors	< 0.26	< 0.26	< 0.26	< 0.35	< 0.35	< 0.35	< 0.35	< 0.3	< 0.3	< 0.71	1
Östrand	Skönviken	< 0.15	< 0.15	< 0.15	< 0.16	< 0.16	< 0.16	< 0.16	< 0.17	< 0.17	< 0.27	1
Östrand	Finsta	< 0.14	< 0.15	< 0.15	< 0.19	< 0.19	< 0.19	< 0.19	< 0.25	< 0.25	< 0.43	1
Östrand	Nacka	< 0.16	< 0.15	< 0.15	< 0.21	< 0.21	< 0.21	< 0.21	< 0.22	< 0.22	< 0.35	1
Iggesund	Byfjärden	< 0.13	< 0.11	< 0.11	< 0.2	< 0.2	< 0.2	< 0.2	< 0.24	< 0.24	< 0.38	1
Iggesund	Gårdsfjärden	< 0.12	< 0.14	< 0.14	< 0.18	< 0.18	< 0.18	< 0.18	< 0.23	< 0.23	< 0.33	1
Iggesund	Mössön	< 0.19	< 0.16	< 0.16	< 0.18	< 0.18	< 0.18	< 0.18	< 0.2	< 0.2	< 0.35	1
Iggesund	Norbergs-fjärden	< 0.1	< 0.12	< 0.12	< 0.11	< 0.11	< 0.11	< 0.11	< 0.11	< 0.11	< 0.18	1
Norrsundet	Norrsundet near	0 .5	< 0.37	< 0.37	< 0.27	< 0.27	< 0.27	< 0.27	< 0.22	< 0.22	< 0.59	1
Norrsundet	Norrsundet remote	0 .5	< 0.3	< 0.3	< 0.24	< 0.24	< 0.24	< 0.24	< 0.24	< 0.24	< 0.55	1
Norrsundet	Axmar Bay	< 0.53	< 0.25	< 0.25	< 0.25	< 0.25	< 0.25	< 0.25	< 0.23	< 0.23	< 0.48	1
Grycksbo	Lake Grycken	3 .3	< 0.051	0 .68	< 0.079	< 0.079	< 0.079	< 0.079	< 0.13	< 0.13	< 0.16	1
Grycksbo	Lake Varpan	< 0.065	< 0.1	< 0.1	< 0.17	< 0.17	< 0.17	< 0.17	< 0.32	< 0.32	< 0.44	1
Grycksbo	Lake Tansen	< 0.056	< 0.11	< 0.11	< 0.12	< 0.12	< 0.12	< 0.12	< 0.19	< 0.19	< 0.14	1
Gruvön	Gruvön	< 0.24	< 0.12	< 0.12	< 0.14	< 0.14	< 0.14	< 0.14	< 0.13	< 0.13	< 0.38	1
Gruvön	Getgar	< 0.2	< 0.23	< 0.23	< 0.21	< 0.21	< 0.21	< 0.21	< 0.31	< 0.31	< 0.65	1
Gruvön	Borgvik	< 0.37	< 0.21	< 0.21	< 0.25	< 0.25	< 0.25	< 0.25	< 0.24	< 0.24	< 0.83	1
Aspa	Aspa near	< 0.3	< 0.18	< 0.18	< 0.35	< 0.35	< 0.35	< 0.35	< 0.46	< 0.46	< 0.88	1
Aspa	Aspa remote	< 0.28	< 0.27	< 0.27	< 0.39	< 0.39	< 0.39	< 0.39	< 0.55	< 0.55	< 1.3	1
Aspa	Bastedalen	< 0.21	< 0.23	< 0.23	< 0.26	< 0.26	< 0.26	< 0.26	< 0.37	< 0.37	< 0.79	1
Comparison site	Holmön	< 0.21	< 0.25	< 0.25	< 0.28	< 0.28	< 0.28	< 0.28	< 0.24	< 0.24	< 0.42	1
Comparison site	Västerås	< 0.27	< 0.3	< 0.3	< 0.21	< 0.21	< 0.21	< 0.21	< 0.3	< 0.3	< 0.56	1
Comparison site	Ängsö	< 0.46	< 0.26	< 0.26	< 0.26	< 0.26	< 0.26	< 0.26	< 0.36	< 0.36	< 1.1	1
Comparison site	Stockholm	0 .33	< 0.094	0 .18	< 0.1	< 0.1	< 0.1	< 0.1	< 0.13	< 0.13	< 0.22	1
Comparison site	Vaxholm	< 0.22	< 0.11	< 0.11	< 0.13	< 0.13	< 0.13	< 0.13	< 0.2	< 0.2	< 0.27	1
Comparison site	Gränna	0 .39	< 0.24	0 .26	< 0.32	< 0.32	< 0.32	< 0.32	< 0.3	< 0.3	< 0.5	1
Comparison site	Gräsö	0 .1	< 0.097	< 0.097	< 0.12	< 0.12	< 0.12	< 0.12	< 0.12	< 0.12	< 0.19	1

a = pooled samples of equal amount of tissue from 4 to 10 individuals

9 and

**Table 10:** dl-PCB levels (pg/g w.w.) in *Perca fluviatilis* of length 15–20 cm

Mill	Sample	PCB 77	PCB 126	PCB 169	PCB 81	PCB 105	PCB 114	PCB 118	PCB 123	PCB 156	PCB 157	PCB 167	PCB 189	n^a^
Obbola	Obbola near	< 6.6	0 .88	< 0.28	< 0.51	57	< 3.4	210	< 3.2	48	7 .1	28	7 .9	1
Obbola	W. Ume River	< 7.6	1 .2	< 0.48	< 0.54	71	< 4.1	240	< 4.8	56	7 .6	34	7 .8	1
Väja	Väja near	< 6.3	0 .9	< 0.27	< 1	67	< 8.7	260	< 5.5	77	12	56	11	1
Väja	Väja remote	< 6.4	1 .1	< 0.4	< 0.62	84	< 8.6	260	< 5.5	52	6 .8	31	3 .6	1
Väja	Sandslån	< 4.5	0 .52	< 0.4	< 0.31	48	< 5.9	140	< 3.1	22	5 .1	15	3 .3	1
Väja	Hallstanäs	< 8.7	0 .88	< 0.48	< 0.44	54	< 7.1	210	< 5.4	38	7 .6	27	6	1
Väja	Kramfors	< 7.8	1 .1	< 0.38	< 0.49	77	< 7	240	< 5.5	56	5 .4	36	13	1
Östrand	Skönviken	< 5.2	0 .79	< 0.16	< 2.4	140	13	480	10	66	5 .5	41	9 .9	1
Östrand	Finsta	< 4.3	< 0.83	< 0.31	< 1.1	62	< 3.5	190	< 1.7	40	7 .6	30	10	1
Östrand	Nacka	< 10	< 0.6	< 0.17	< 2.8	77	< 3.6	240	< 3	33	3 .4	15	5 .8	1
Iggesund	Byfjärden	< 9.5	0 .77	< 0.17	2 .5	92	< 3.6	290	1 .9	47	6 .7	28	4 .1	1
Iggesund	Gårdsfjärden	< 5.7	1 .2	< 0.11	1 .2	89	< 3.6	230	2 .5	26	4 .2	19	6 .3	1
Iggesund	Mössön	< 2.7	1	< 0.13	1 .1	66	< 0.52	200	< 1.4	29	2 .6	17	5 .1	1
Iggesund	Norbergsfjärden	< 3.6	< 0.46	< 0.15	< 0.88	61	< 2	200	< 1.5	32	4 .9	14	3 .3	1
Norrsundet	Norrsundet near	< 5.7	0 .81	< 0.19	< 0.38	48	< 2.8	180	< 4.7	47	9 .3	25	7 .6	1
Norrsundet	Norrsundet remote	< 8.7	1 .1	< 0.18	< 0.47	48	< 2.9	160	< 3.4	35	5 .9	20	5 .5	1
Norrsundet	Axmar Bay	< 7.2	0 .54	< 0.19	< 0.34	< 43	< 4.3	120	< 1.6	25	3 .6	22	6 .1	1
Grycksbo	Lake Grycken	< 5.3	1 .9	< 1.4	< 3.9	120	32	640	4 .3	300	23	140	37	1
Grycksbo	Lake Varpan	< 3.7	2 .2	< 1.4	< 8.5	73	16	340	4 .9	180	17	98	25	1
Grycksbo	Lake Tansen	< 6.3	< 4.3	< 3.6	< 12	52	< 3.5	140	< 0.83	59	6 .6	30	6 .5	1
Gruvön	Gruvön	< 10	1 .2	< 0.17	< 0.52	140	8 .8	540	4 .3	140	13	51	11	1
Gruvön	Getgar	< 5.7	< 0.76	< 0.59	< 0.6	< 43	< 3.5	160	< 2.4	30	4 .7	22	4 .5	1
Gruvön	Borgvik	< 5.6	1 .6	< 0.52	< 0.48	67	< 3.6	220	< 2.5	60	5 .2	53	13	1
Aspa	Aspa near	< 7.4	1 .8	< 0.34	< 1.2	56	< 3.7	170	< 3.4	44	7 .3	32	9 .2	1
Aspa	Aspa remote	< 7.5	2 .5	< 0.4	< 0.74	70	< 4.1	230	< 4.1	76	11	43	10	1
Aspa	Bastedalen	< 9.6	2 .9	< 0.47	< 3.3	94	6 .2	300	5 .6	88	13	62	15	1
Comparison site	Holmön	< 4.9	0 .71	< 0.18	< 0.37	< 42	< 4	110	< 4.8	26	5 .9	21	5 .1	1
Comparison site	Västerås	< 16	3 .9	< 0.62	< 0.81	300	6 .7	920	8 .9	210	38	160	23	1
Comparison site	Ängsö	< 11	1 .3	< 0.75	< 0.56	150	7 .1	500	8 .2	99	13	71	16	1
Comparison site	Stockholm	59	5 .6	< 1.2	5 .2	1200	27	3500	60	680	100	300	51	1
Comparison site	Vaxholm	< 17	1 .9	< 1.6	< 2.9	290	18	1200	24	200	34	110	22	1
Comparison site	Gränna	< 12	5 .2	< 0.64	< 0.85	59	11	300	6 .5	94	< 68	59	< 58	1
Comparison site	Gräsö	< 4.6	0 .64	< 0.42	< 1	< 43	< 1.6	130	< 2	32	< 2.1	19	< 2.2	1

a = pooled samples of equal amount of tissue from 4 to 10 individuals

10

## Appendix C: Morphometric Variables

See Tables

**Table 11:** Length, weight and condition factor (CF) in *Perca fluviatilis*. mean ± se

Mill	Site	Sampling date	Length (cm)	Weight (g)	Age (years)	Growth at age (cm/year)	CF	n
Obbola	Obbola near	2018–11-15	17.6 ± 0.174	58.8 ± 1.71	3.04 ± 0.105	6.06 ± 0.209	0.956 ± 0.0102	45
Obbola	W. Ume River	2018–11-15	17.9 ± 0.275	66.5 ± 3.1	2.71 ± 0.165	7.09 ± 0.364	1.01 ± 0.0137	24
Dynäs	Dynäs near	2019–09-03	17.6 ± 0.118	57.9 ± 1.35	4.53 ± 0.128	4.08 ± 0.0996	0.981 ± 0.00809	72
Dynäs	Dynäs remote	2019–09-03	17.6 ± 0.169	57.2 ± 1.62	4.36 ± 0.141	4.2 ± 0.113	0.965 ± 0.00867	55
Dynäs	Sandslån	2019–09-03	17.3 ± 0.101	61.7 ± 1.16	4.36 ± 0.0856	4.12 ± 0.0713	1.07 ± 0.0076	119
Hallstanäs	Hallstanäs	2019–09-04	18 ± 0.218	61.8 ± 2.31	5.55 ± 0.226	3.48 ± 0.138	0.971 ± 0.0142	45
Kramfors	Kramfors	2019–09-04	17.7 ± 0.147	61.5 ± 1.53	5.0 ± 0.167	3.76 ± 0.0993	1.01 ± 0.0104	74
Östrand	Finsta	2017–09-07	18.2 ± 0.288	80.1 ± 4.95	4.47 ± 0.192	4.17 ± 0.159	1.16 ± 0.0244	15
Östrand	Nacka	2017–09-07	16.7 ± 0.232	57.2 ± 2.7	3.6 ± 0.190	4.78 ± 0.218	1.09 ± 0.0208	15
Östrand	Skönviken	2017–09-14	17.7 ± 0.479	72.2 ± 7.65	4.0 ± 0.196	4.52 ± 0.186	1.12 ± 0.026	13
Iggesund	Byfjärden	2017–10-25	18.7 ± 0.379	79.6 ± 6.23	4.12 ± 0.241	4.74 ± 0.213	1.05 ± 0.0335	17
Iggesund	Gårdsfjärden	2017–10-25	18.6 ± 0.433	77.2 ± 6.04	4.25 ± 0.194	4.47 ± 0.169	1.01 ± 0.019	16
Iggesund	Mössön	2017–10-26	17.6 ± 0.356	66.3 ± 4.59	4.12 ± 0.272	4.49 ± 0.253	1.07 ± 0.0212	16
Iggesund	Norbergsfjärden	2017–10-23	18.1 ± 0.456	69.9 ± 6.09	4.29 ± 0.268	4.41 ± 0.227	0.991 ± 0.0246	17
Norrsundet	Axmar Bay	2018–11-15	17.9 ± 0.357	73.1 ± 4.62	2.0 ± 0.114	9.5 ± 0.651	1.14 ± 0.0162	18
Norrsundet	Norrsundet near	2018–11-15	19 ± 0.237	91.6 ± 3.83	1.92 ± 0.0833	10.4 ± 0.554	1.2 ± 0.0143	24
Norrsundet	Norrsundet remote	2018–11-15	16.9 ± 0.94	68.2 ± 9.75	3.0 ± 0	5.63 ± 0.313	1.26 ± 0.0409	4
Grycksbo	Lake Grycken	2019–09-22	17.8 ± 0.249	57.1 ± 2.96	4.41 ± 0.187	4.24 ± 0.195	0.918 ± 0.00999	27
Grycksbo	Lake Tansen	2019–09-22	18.1 ± 0.181	62 ± 2.65	4.59 ± 0.203	4.24 ± 0.207	0.927 ± 0.0122	37
Grycksbo	Lake Varpan	2019–10-14	18 ± 0.235	57 ± 2.75	4.29 ± 0.223	4.49 ± 0.215	0.89 ± 0.011	28
Gruvön	Borgvik	2018–09-13	17.7 ± 0.28	61.1 ± 3.42	4.0 ± 0.178	4.55 ± 0.158	0.959 ± 0.0234	20
Gruvön	Getgar	2018–09-11	17.7 ± 0.282	63 ± 3.03	3.9 ± 0.143	4.63 ± 0.15	1.03 ± 0.0235	20
Gruvön	Gruvön	2018–09-11	18.4 ± 0.264	73 ± 2.96	4.0 ± 0.145	4.71 ± 0.146	1.05 ± 0.0168	20
Aspa	Aspa near	2019–09-19	17.7 ± 0.144	59.2 ± 1.51	2.7 ± 0.0915	6.87 ± 0.201	0.975 ± 0.0109	50
Aspa	Aspa remote	2019–09-18	17.9 ± 0.148	61.5 ± 1.74	2.6 ± 0.10	7.35 ± 0.238	0.974 ± 0.0112	52
Aspa	Bastedalen	2019–09-19	18.5 ± 0.265	71.2 ± 3.24	2.85 ± 0.134	6.88 ± 0.268	1.01 ± 0.0121	35

^11^ and

**Table 12:** Liver somatic index (LSI) and gonadosomatic index (GSI) in *Perca fluviatilis*. mean ± se

Mill	Site	Sampling date	LSI female	LSI male	GSI female	GSI male	n LSI female^a^	n LSI male^a^	n GSI female^b^	n GSI male^b^
Obbola	Obbola near	2018–11-15	1.18 ± 0.0625	0.894 ± 0.043	3.18 ± 0.439	9.48 ± 0.425	16	28	6	28
Obbola	W. Ume River	2018–11-15	1.4 ± 0.0916	1.04 ± 0.0568	4.21 ± 1.16	11.2 ± 0.491	13	11	2	11
Dynäs	Dynäs near	2019–09-03	0.647 ± 0.0311	0.539 ± 0.0305	2.77 ± 0.275	6.03 ± 0.573	52	19	13	15
Dynäs	Dynäs remote	2019–09-03	0.697 ± 0.0441	0.519 ± 0.0419	2.95 ± 0.316	5.42 ± 0.529	35	20	14	18
Dynäs	Sandslån	2019–09-03	1.44 ± 0.0652	1.35 ± 0.0687	2.35 ± 0.103	6.87 ± 0.374	74	45	36	44
Hallstanäs	Hallstanäs	2019–09-04	0.664 ± 0.0332	0.532 ± 0.066	3.32 ± 0.121	5.65 ± 0.667	35	7	14	7
Kramfors	Kramfors	2019–09-04	0.742 ± 0.0286	0.609 ± 0.0363	2.52 ± 0.219	6.97 ± 0.704	56	16	18	13
Östrand	Finsta	2017–09-07	1.75 ± 0.136	1.32 ± 0.182	1.37 ± 0.213	3.73 ± 0.392	12	3	2	2
Östrand	Nacka	2017–09-07	1.53 ± 0.109	1.48 ± 0.16	-	5.5 ± 0.898	10	4	0	4
Östrand	Skönviken	2017–09-14	1.43 ± 0.0986	1.23 ± 0.152	1.95 ± 0.215	4.46 ± 1.04	7	6	2	5
Iggesund	Byfjärden	2017–10-25	1.5 ± 0.145	1.2 ± 0.106	5.62 ± 1.5	6.45 ± 0.818	11	6	4	6
Iggesund	Gårdsfjärden	2017–10-25	2.14 ± 0.211	1.55 ± 0.0984	7.97 ± 1.99	6.8 ± 0.278	7	9	3	9
Iggesund	Mössön	2017–10-26	1.59 ± 0.103	1.49 ± 0.104	7.06	6.37 ± 0.25	8	8	1	8
Iggesund	Norbergsfjärden	2017–10-23	1.83 ± 0.29	1.27 ± 0.0637	8.93 ± 0.71	7.24 ± 0.256	4	13	2	13
Norrsundet	Axmar Bay	2018–11-15	1.55 ± 0.082	1.61 ± 0.167	4.22 ± 1.14	7.22 ± 1.91	13	5	2	2
Norrsundet	Norrsundet near	2018–11-15	1.73 ± 0.0681	1.68 ± 0.144	-	7.02 ± 0.967	17	7	0	4
Norrsundet	Norrsundet remote	2018–11-15	1.78 ± 0.145	1.99	4.72 ± 1.26	-	3	1	2	0
Grycksbo	Lake Grycken	2019–09-22	1.17 ± 0.0671	0.829	2.84 ± 0.189	8.03 ± NA	26	1	20	1
Grycksbo	Lake Tansen	2019–09-22	1.71 ± 0.118	1.5	2.73 ± 0.172	11.9	36	1	26	1
Grycksbo	Lake Varpan	2019–10-14	1.02 ± 0.0753	0.825 ± 0.146	2.41 ± 1.62	6.15 ± 0.622	26	2	3	2
Gruvön	Borgvik	2018–09-13	1.04 ± 0.131	0.646 ± 0.0548	4.38 ± 0.0443	3.81 ± 0.658	10	10	2	9
Gruvön	Getgar	2018–09-11	0.796 ± 0.045	0.826 ± 0.116	-	2.97 ± 0.572	14	6	0	6
Gruvön	Gruvön	2018–09-11	1.03 ± 0.0916	0.728 ± 0.0462	1.94 ± 0.338	3.63 ± 0.35	11	9	3	9
Aspa	Aspa near	2019–09-19	1.07 ± 0.0603	0.991 ± 0.0512	2.2	5.08 ± 0.525	30	20	1	16
Aspa	Aspa remote	2019–09-18	0.987 ± 0.0523	0.9 ± 0.0733	2.51 ± 0.457	4.9 ± 0.548	36	16	6	14
Aspa	Bastedalen	2019–09-19	1.08 ± 0.0475	0.931 ± 0.0447	2.66 ± 0.13	5.69 ± 0.829	25	10	4	9

a = juvenile + adult (sexually mature) individuals. b = only adult individuals

12.

## Declarations

### Availability of Data and Material

All data are published in Appendices.

### Code Availability

An example code of the statistical analysis performed in R (R Core Team 2020) is available in Waldetoft et al. (2020).
